# Drug Repurposing: Conversion of the Peripherally Restricted HIV Protease Inhibitor Amprenavir to Potent, Selective, and CNS-Penetrant Agonists for the Cannabinoid Receptor 2

**DOI:** 10.1021/acs.jmedchem.5c02796

**Published:** 2026-02-05

**Authors:** Daniel H. Haymer, Renn A. Duncan, Alice L. Rodriguez, Allie Han, Richard J. Lindsay, N. Kithmini Wijesiri, Analisa Thompson Gray, Srinivasan Krishnan, Aidong Qi, Benjamin P. Brown, Olivier Boutaud, Darren W. Engers, Carrie K. Jones, Colleen M. Niswender, Craig W. Lindsley, Aaron M. Bender

**Affiliations:** a Warren Center for Neuroscience Drug Discovery, 5718 Vanderbilt University, Nashville, Tennessee 37232, United States; b Department of Pharmacology, 5718 Vanderbilt University, Nashville, Tennessee 37232, United States; c Center for AI in Protein Dynamics, 5718 Vanderbilt University, Nashville, Tennessee 37232, United States; d Vanderbilt Brain Institute, 5718 Vanderbilt University, Nashville, Tennessee 37232, United States; e Vanderbilt Kennedy Center, 12328 Vanderbilt University Medical Center, Nashville, Tennessee 37232, United States; f Vanderbilt Institute of Chemical Biology, 5718 Vanderbilt University, Nashville, Tennessee 37232, United States; g Department of Chemistry, 5718 Vanderbilt University, Nashville, Tennessee 37232, United States; h Vanderbilt Institute for Therapeutic Advances, 5718 Vanderbilt University, Nashville, Tennessee 37232, United States

## Abstract

Herein, we report the identification of the HIV protease inhibitor amprenavir as a selective cannabinoid receptor 2 (CB_2_) agonist and describe structure–activity relationship (SAR) studies toward repurposing this peripherally restricted scaffold for high CB_2_ potency and CNS exposure. This exercise yielded compounds with exceptional CB_2_ potency (EC_50_s <10 nM), no appreciable activity at the CB_1_ receptor, and high predicted permeability/low P-gp efflux activity. Selected compounds were profiled in rat i.v. dosing cassettes; several novel amprenavir analogues displayed good *t*_1/2_ (>2 h), moderate plasma clearance, and appreciable brain exposure. Additionally, fully flexible protein–ligand docking studies with molecular dynamics (MD) simulations were used to predict the most likely mode of interaction of highly potent analogue VU6077967 with CB_2_ and to provide a rationale for the observed selectivity of this series relative to CB_1_.

## Graphical abstract

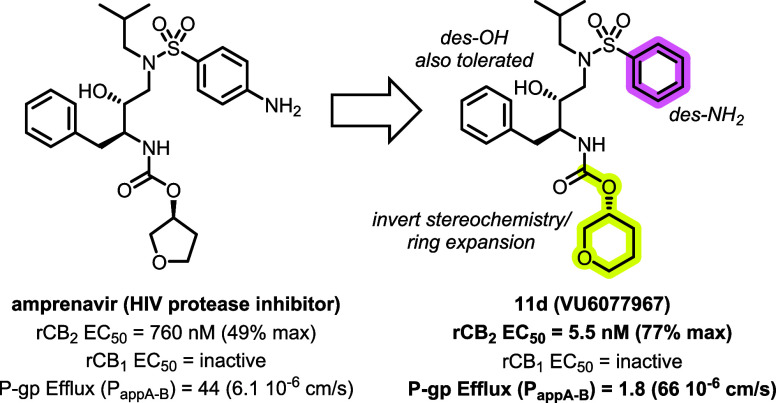

## Introduction

The identification of the cannabinoid receptor 1 (CB_1_) as the primary driver for the *Cannabis*-derived phytocannabinoid psychoactivity has led to considerable interest in the druggability of the endocannabinoid (EC) system.^1^ CB_1_ and the cannabinoid receptor 2 (CB_2_) are well-characterized receptors responsible for the effects of the cannabinoid pharmacopoeia (the phytocannabinoids, including (−)-trans-Δ^9^-tetrahydrocannabinol (THC) and related compounds, synthetic THC derivatives, and nonphytocannabinoid chemotypes). CB_1_ and CB_2_ are G protein-coupled receptors (GPCRs) that primarily couple to G_i/o_ proteins, activation of which leads to decreased adenylyl cyclase activity, reduced cyclic adenosine monophosphate (cAMP) production, and ion channel modulation. The primary ECs for CB_1_ and CB_2_ are the cell membrane phospholipid-derived anandamide (AEA) and 2-arachidonoyl-glycerol (2-AG).^2–4^ To date, the interactions of AEA and 2-AG with CB_1_ and CB_2_ account for the majority of research on the EC system, although additional endogenous and exogenous cannabinoids are known to interact with other closely homologous orphan GPCRs.^5,6^

CB_1_ is highly expressed in the central nervous system (CNS), with the highest tissue expression levels in the basal ganglia, hippocampus, and cerebellum.^7^ CB_2_, by contrast, is predominantly expressed in peripheral tissues.^8,9^ These relative tissue distribution patterns have historically pigeonholed CB_1_ and CB_2_ as the “central” and “peripheral” cannabinoid receptors, but an increased understanding of their distribution has refined this picture. CB_1_ is also found in peripheral tissues, and advances in protein detection have indicated the presence of CB_2_ across a diverse range of brain regions (albeit in lower abundance relative to CB_1_).^7–9^ This broad distribution of CB_2_ in the CNS, including the hippocampus, cerebral cortex, striatum, olfactory and spinal nuclei, amygdala, thalamus, and cerebellum,^9,10^ has spurred a wealth of exciting research clarifying the receptor’s role across a variety of CNS-based indications. Indeed, the receptor and its ligands have been studied in the context of Alzheimer’s disease,^11^ Huntington’s disease,^12^ Parkinson’s disease,^13^ multiple sclerosis,^14^ and emotional disorders^15^ (in addition to a diverse set of peripheral indications related to inflammation, pain, and autoimmune disorders).^4^ Additionally, our laboratories have shown that the antipsychotic activity of muscarinic acetylcholine receptor 4 (M_4_) activators requires intact CB_2_ signaling, suggesting that CB_2_ is a potential therapeutic target for schizophrenia and related psychoses.^16^

Although structurally diverse CB_2_ modulators have entered clinical development (see Figure 1 for selected examples),^17–20^ to date, no CB_2_-selective compound has reached the market.^3^ While much remains to be learned from these trials and many laboratories continue to make exciting progress in the development of drug-like CB_2_ compounds,^21–28^ this lack of success suggests a need for CB_2_ ligands with improved on-target engagement, superior physicochemical properties, and/or higher selectivity relative to CB_1_ (e.g., lower lipophilicity and target promiscuity relative to the THC-type exocannabinoids). Drug development for CB_2_ is further confounded by the complexities of the receptor’s signaling and molecular pharmacology. Although CB_2_ is classically G_i/o_-coupled, instances of G_αq_ and G_αs_ coupling have also been observed.^29–31^ Additionally, differences in functional selectivity (signaling bias) have been observed across CB_2_ chemotypes and across preclinical species.^3,32,33^ Recently, we reported this type of mixed CB_2_ pharmacology for several structurally unique CB_2_ agonists and further characterized a range of binding interactions at the canonical orthosteric 2-AG pocket for these probes.^34,35^ Indeed, computational studies have indicated that CB_2_ may possess up to seven druggable binding pockets, raising important questions about probe dependence and functional selectivity in preclinical assays.^36^ Clearly, there exists an unmet need for the detailed pharmacological characterization of new and existing CB_2_ chemotypes.

**Figure 1:**
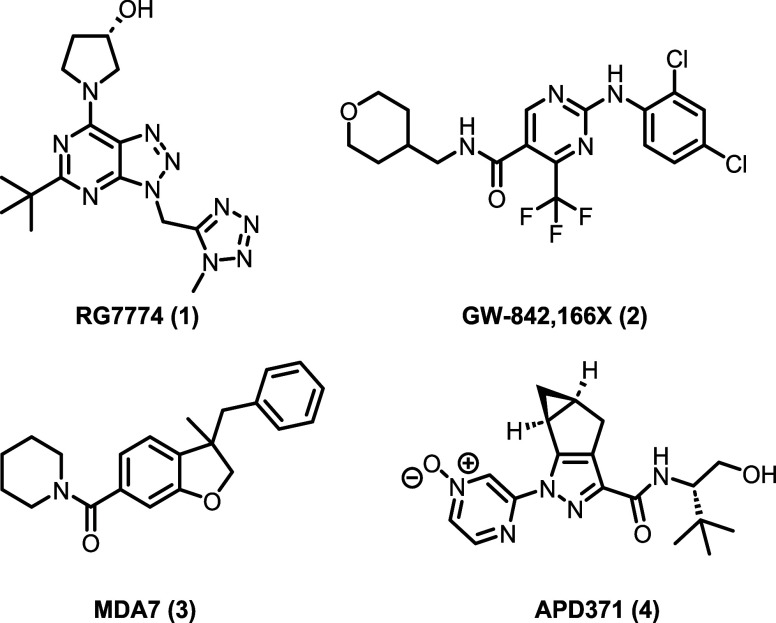
Chemical structures of selected clinical CB_2_ agonists. See refs 17–20 .

To identify such chemotypes, our laboratory recently screened a small library (∼1000 compounds) of FDA-approved drugs for novel CB_2_ chemical matter using a thallium flux-based functional assay as a pilot for a larger high-throughput screen (HTS). In addition to the identification of the immunosuppressant mycophenolate mofetil as a potent and selective CB_2_ activator,^35^ this screen also identified the HIV protease inhibitor amprenavir^37^ (Figure 2A) as a CB_2_ activator (EC_50_ = 760 nM, 49% Max in the thallium flux assay in rat CB_2_/G protein-coupled inwardly rectifying potassium channel (GIRK) cells; see Table 1). Intrigued by this unusual finding and the prospect of repurposing an antiviral compound for a GPCR in the CNS, we sought to understand the structure–activity relationship (SAR) requirements of the amprenavir scaffold for CB_2_ activity. At the outset of our SAR campaign, we were aware of several challenges associated with this endeavor, namely, the difficulty in attaining reasonable CNS exposure for such a chemotype (amprenavir is a large, polar molecule and a known substrate for P-glycoprotein (P-gp)-mediated efflux).^38^ Nevertheless, we designed our initial analogs with the goal of improving the CNS penetration of the amprenavir chemotype.

**Figure 2:**
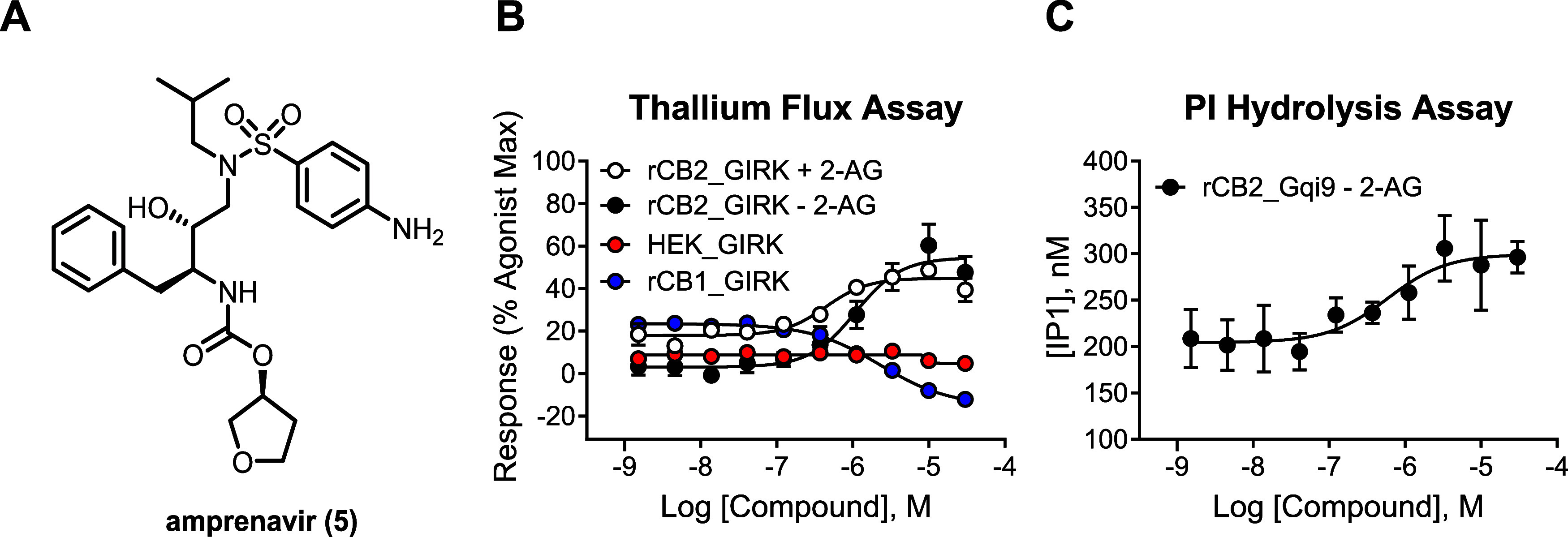
Chemical structure (A), thallium flux (B), and (C) PI hydrolysis data for amprenavir (**5**). For thallium flux, data represent the compound profile in rat CB_2_/GIRK cells run in the presence (white circles) and absence (black circles) of a submaximal concentration of 2-AG. No significant response was noted in non-CB_2_-expressing HEK cells (red circles). Amprenavir (**5**) does not activate rCB_1_ and appears to function as a weak inhibitor in this cell line (blue circles). For PI hydrolysis, data represent compound in rat CB_2_/G_qi9_ cells run in the absence (black circles) of a submaximal concentration of 2-AG.

**Table 1:**
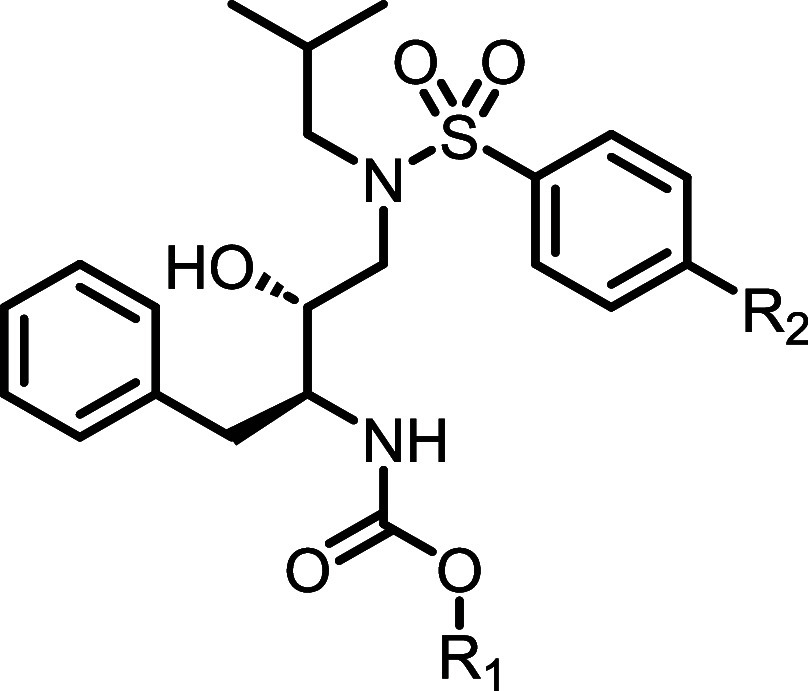

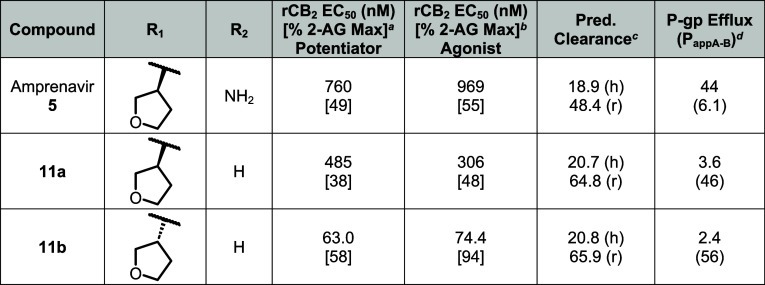
CB_2_ Potency, Predicted Hepatic Clearance, and P-gp Efflux Data for Compounds **5** and **11a**,**b** ^a^

a Thallium flux assays in rat CB_2_/GIRK cells run in (a) the presence and (b) absence of submaximal 2-AG. EC_50_ values are calculated from the mean pEC_50_ values of at least three independent experiments run in duplicate or triplicate unless otherwise noted. % 2-AG Max represents the maximal compound response as a percentage of 100 μM 2-AG. (c) Predicted hepatic clearance (microsomes), h = human, r = rat. (d) Efflux ratios for compounds tested in human P-glycoprotein (P-gp)-transfected MDCKII-MDR1 cells. *P*_appA‑B_ is 10^–6^ cm/s.

## Results and Discussion

Encouraged by the identification of amprenavir (**5**) as a novel CB_2_ activator, we first confirmed that amprenavir exerts its activity through CB_2_ by testing it in HEK cells expressing GIRK channels but not CB_2_ and found it to be inactive under those conditions, suggesting CB_2_-dependent activity (Figure 2B, red circles). We also determined subtype selectivity by testing amprenavir activity in cells expressing rCB_1_ and GIRK channels where it did not enhance the response but instead inhibited it (Figure 2B, blue circles). As our thallium flux assay is routinely run in the presence of a submaximal concentration of 2-AG to allow for detection of potentiators, it was important to also determine the activity of amprenavir in the absence of 2-AG. In this context, amprenavir (**5**) potency was found to be comparable (EC_50_ = 969 nM, 55% Max, Figure 2B, black circles) to that determined in the presence of 2-AG (Figure 2B, white circles), consistent with agonist activity as opposed to potentiator activity. We confirmed agonist activity (EC_50_ = 690 nM) in an orthogonal PI hydrolysis assay coupled via a chimeric G protein in the absence of 2-AG (Figure 2C). We next examined the predicted clearance of **5** in human and rat microsomes, as well as the predicted human P-gp efflux in transfected MDCKII-MDR1 cells, to understand its pharmacokinetic (PK) properties. Amprenavir (**5**) was found to have high predicted hepatic clearance in microsomes for both species and was reconfirmed as a substrate for P-gp efflux (efflux ratio = 44; *P*_appA‑B_ = 6.1 × 10^–6^ cm/s, see Table 1). As such, we identified three major areas for scaffold improvement: (1) improvement of CB_2_ agonist potency, (2) improvement of predicted microsomal clearance across species (human/rat), and (3) reduction in predicted P-gp efflux.

Surmising that the aniline motif in amprenavir (**5**) was at least partially responsible for the observed limited brain exposure, we first synthesized the direct *des*-NH_2_ analog of amprenavir, compound **11a** (Table 1). Compound **11a**, along with additional analogues in the *des*-NH_2_ series, were synthesized as shown in Scheme 1. Commercially available secondary amine **6** was first protected as the benzyl carbamate to give **7**, from which Boc-deprotection gave **8** as the HCl salt. Intermediate **8** could then be elaborated to the desired carbamates **9** (in the case of **11a**, the (*S*)-3-furanyl derivative). Following Cbz deprotection, intermediates **10** were coupled with the desired benzenesulfonyl chloride to give final compounds **11a** and **14a**–**i**. Additional final analogues (**11b**–**j**) were synthesized in a similar fashion (Scheme 2), with installation of the benzenesulfonamide preceding Boc deprotection and southern pendant elaboration.^39,40^

**Scheme 1:**
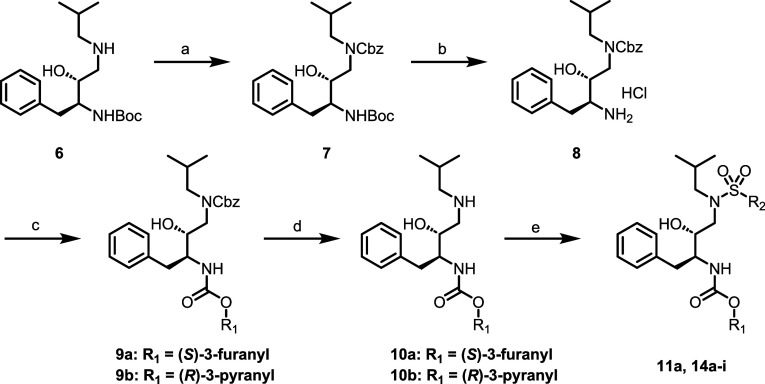
Synthesis of Compounds **11a** and **14a**–**i** ^a^

a Reagents and conditions: (a) benzyl chloroformate, K_2_CO_3_, THF, H_2_O, 0 °C–r.t., 67%; (b) HCl, 1,4-dioxane, r.t., 100%; (c) substituted alcohol, disuccinimidyl carbonate, pyridine, TEA, MeCN, r.t.; (d) Pd­(OH)_2_, H_2_, MeOH, r.t., 98%; (e) substituted sulfonyl chloride, NaHCO_3_, DCM, H_2_O, 0 °C–r.t., 18–72%.

**Scheme 2:**
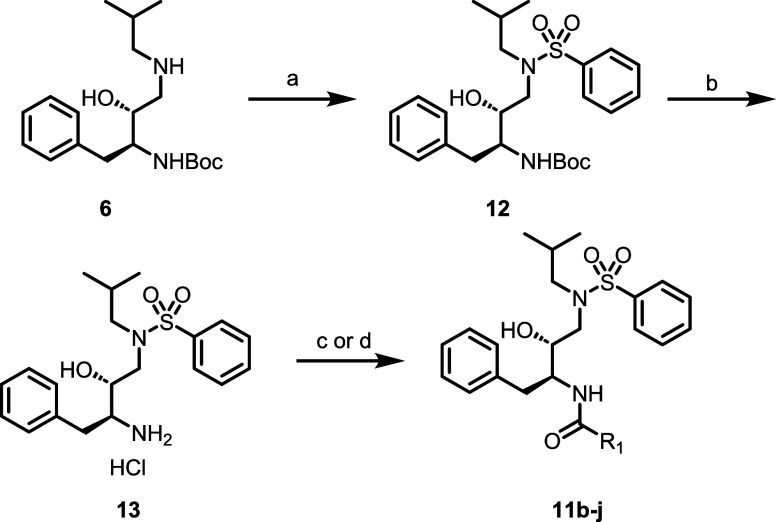
Synthesis of Compounds **11b**–**j** ^a^

a Reagents and conditions: (a) benzenesulfonyl chloride, NaHCO_3_, DCM, H_2_O, 0 °C–r.t., 64%; (b) HCl, 1,4-dioxane, r.t., 95%; (c) substituted alcohol, disuccinimidyl carbonate, pyridine, TEA, MeCN, r.t., 39–66%; (d) substituted carboxylic acid, HATU, DIPEA, DCM, r.t., 41–82%.

Encouragingly, the direct *des*-aniline analogue **11a** was found to have improved agonist potency relative to amprenavir in the absence of 2-AG (CB_2_ EC_50_s = 306 and 969 nM, respectively), as well as a dramatic improvement in P-gp efflux (efflux ratio = 3.6; *P*_appA‑B_ = 46 × 10^–6^ cm/s). We were further gratified to find that the opposite enantiomer furan ((*R*)-furanyl analogue **11b**) displayed an even greater improvement in potency (74 nM, roughly an 4× potency increase relative to **11a**), along with low predicted P-gp efflux activity and high passive permeability (efflux ratio = 2.4; *P*_appA‑B_ = 56 × 10^–6^ cm/s; see Table 1).

With the understanding that the aniline motif of **5** was detrimental with respect to potency and efflux, we next turned our attention to a survey of the southern carbamate pendant in the context of the unsubstituted benzenesulfonamide (analogues **11c**–**j**; see Table 2). Chiral 3-pyranyl compounds **11c** and **11d** were found to be more potent than the 3-furanyl counterparts (**11a** and **11b**) and followed a similar trend with respect to enantiopreference. Indeed, (*R*)-pyranyl analogue **11d** was approximately 4-fold more potent than (*S*)-pyranyl analogue **11c** and was the most potent analogue synthesized in the series thus far (CB_2_ EC_50_ = 8.6 nM). Interestingly, 4-pyranyl analogue **11e** and cyclobutyl analogue **11g** did not activate the receptor but instead induced a decrease in response. Potency was also reduced in the case of cyclopropyl analogue **11f**. Conversely, we were encouraged to find that additional aliphatic groups were well tolerated when switching from a carbamate pendant to an amide, including bicyclo[1.1.1]­pentane **11h**, cyclopropane-1-carboxamide **11i** and cyclobutane-1-carboxamide **11j**. In general, within the amide series, the presence of a quaternary carbon adjacent to the carbonyl was well tolerated and tended to yield highly potent analogues. Efficacy values ranged from 36 to 130% 2-AG Max and continued to demonstrate partial to full agonist activity.

**Table 2:**
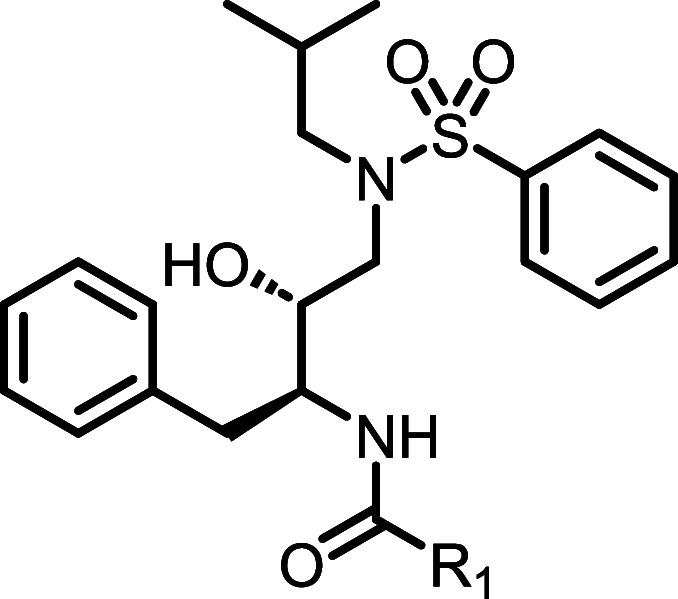

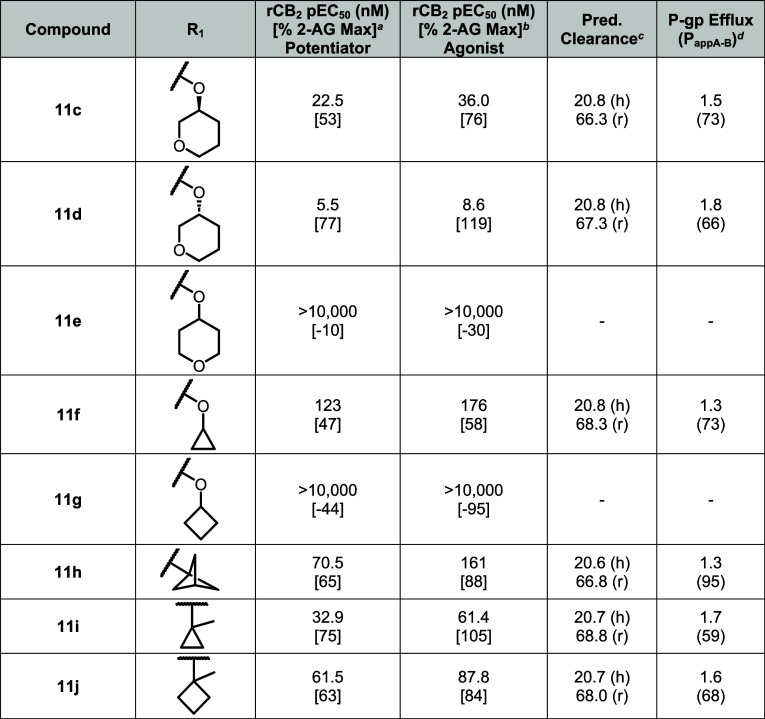
CB_2_ Potency, Predicted Hepatic Clearance, and P-gp Efflux Data for Compounds **11c**–**j** ^a^

a Thallium flux assays in rat CB_2_/GIRK cells run in (a) the presence and (b) absence of submaximal 2-AG. EC_50_ values are calculated from the mean pEC_50_ values of at least three independent experiments run in duplicate or triplicate unless otherwise noted. % 2-AG Max represents the maximal compound response as a percentage of 100 μM 2-AG. (c) Predicted hepatic clearance (microsomes), h = human, r = rat. (d) Efflux ratios for compounds tested in human P-glycoprotein (P-gp)-transfected MDCKII-MDR1 cells. *P*_appA‑B_ is 10^–6^ cm/s. “–” indicates data was not collected.

Given the exceptional potency of (*R*)-pyranyl analogue **11d**, we next held this southern carbamate pendant constant while surveying changes to the sulfonamide (Table 3). A variety of sulfonamides were well tolerated in this context, including several modifications to the *para*-position (**14a**–**c**), as well as a variety of heteroaromatics (**14e**–**h**). While diverse sulfonamides were well tolerated in general, some exceptions were noted: (1) aliphatic sulfonamides tended to be markedly less potent (see cyclopropyl analogue **14d**), and (2) modifications to the *ortho* position were often detrimental (see thiazole analogue **14i**). Additional modifications to the sulfonamide motif that abolished activity were (1) replacement with an amide or amine and (2) cyclization of the nucleophilic chiral secondary hydroxy group to give a 2-substituted 3,4-dihydro-2*H*-benzo­[*b*]­[1,4,5]­oxathiazepine 1,1-dioxide system.

**Table 3:**
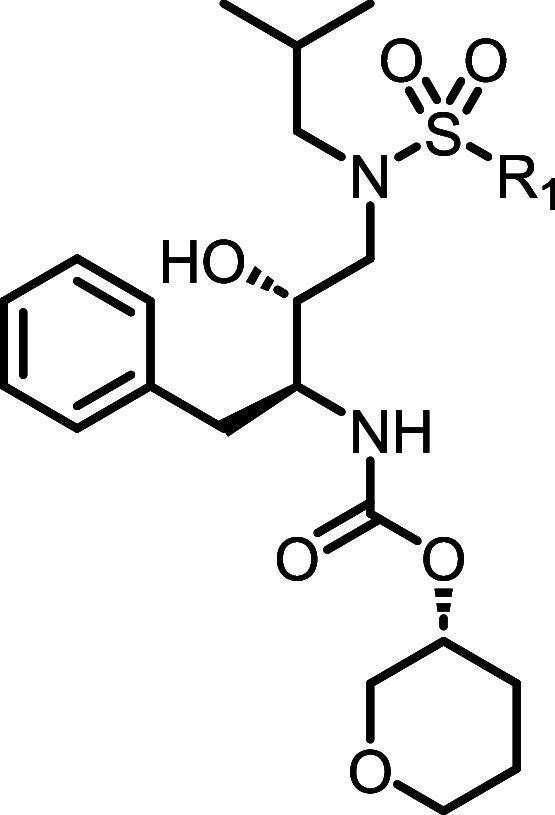

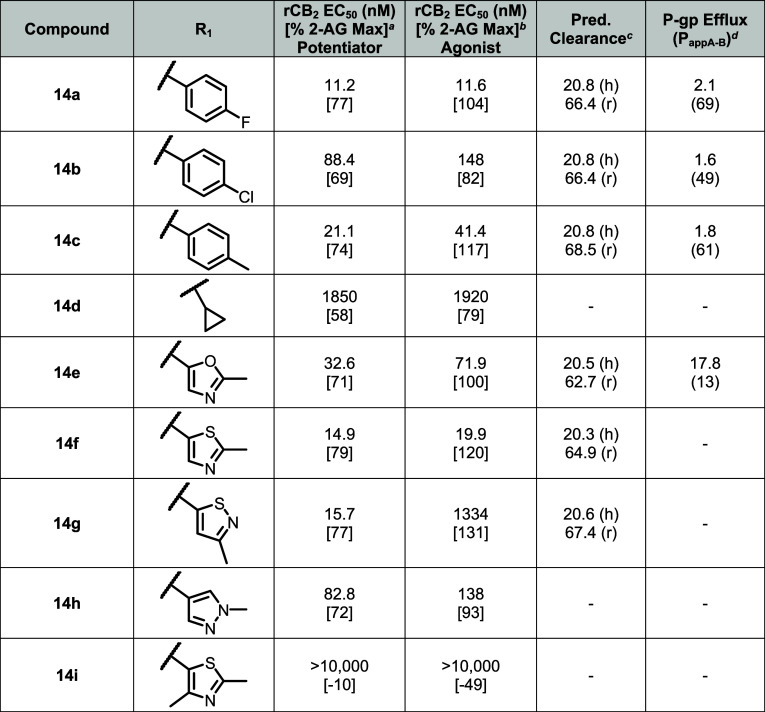
CB_2_ Potency, Predicted Hepatic Clearance, and P-gp Efflux Data for Compounds **14a**–**i** ^a^

a Thallium flux assays in rat CB_2_/GIRK cells run in (a) the presence and (b) absence of submaximal 2-AG. EC_50_ values are calculated from the mean pEC_50_ values of at least three independent experiments run in duplicate or triplicate unless otherwise noted. % 2-AG Max represents the maximal compound response as a percentage of 100 μM 2-AG. (c) Predicted hepatic clearance (microsomes), h = human, r = rat. (d) Efflux ratios for compounds tested in human P-glycoprotein (P-gp)-transfected MDCKII-MDR1 cells. *P*_appA‑B_ is 10^–6^ cm/s. “–” indicates data were not collected.

Unfortunately, although P-gp efflux remained low for the majority of analogues, all compounds surveyed to this point were found to have similarly high predicted microsomal clearance for both human and rat, suggesting that alternative modifications would be necessary to address the metabolic hotspot(s) for this scaffold.

To this end, we next examined the deletion of the chiral secondary hydroxyl group; final analogues in the *des*-hydroxy series were prepared as shown in Scheme 3. Briefly, commercially available Boc-protected β-amino acid **15** was first reduced to give primary alcohol **16**, which was oxidized under Parikh-Doering conditions to give aldehyde **17**. Reductive amination with isobutylamine gave secondary amine **18**, from which intermediate **20** was generated after sulfonamide formation and Boc deprotection. As before, **20** was coupled with the desired alcohols or carboxylic acids to give carbamate and amide analogues **21**.

**Scheme 3:**
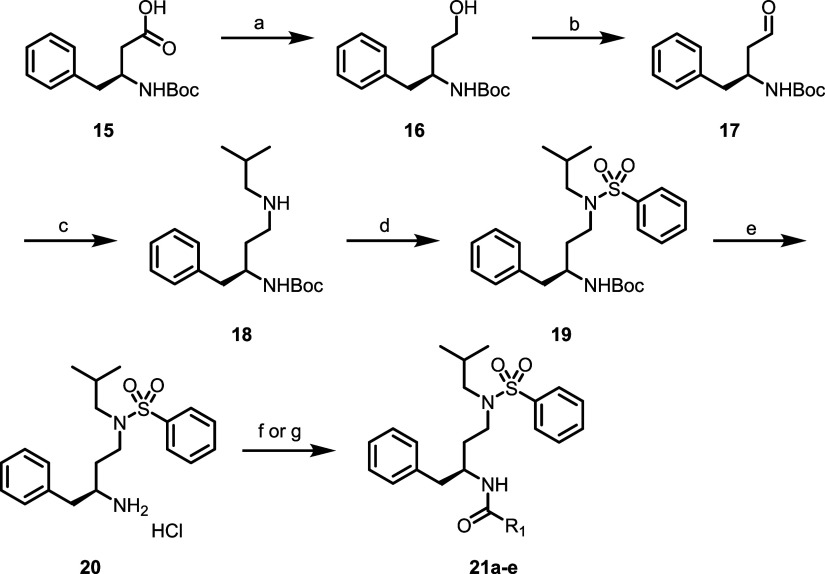
Synthesis of Compounds **21a**–**e** ^a^

a Reagents and conditions: (a) LAH, THF, 0 °C–r.t., 87%; (b) sulfur trioxide pyridine complex, DIPEA, DMSO, 0 °C–r.t., 100%; (c) isobutylamine, NaBH­(OAc)_3_, DCM, r.t., 88%; (d) benzenesulfonyl chloride, NaHCO_3_, DCM, H_2_O, 0 °C–r.t., 87%; (e) HCl, 1,4-dioxane, r.t., 100%; (f) substituted alcohol, disuccinimidyl carbonate, pyridine, TEA, MeCN, r.t., 38–49%; (g) substituted carboxylic acid, HATU, DIPEA, DMF, r.t., 47–63%.

In general, although potency within the *des*-hydroxy series was steeper relative to the previous series, certain carbamate/amide pendants were well tolerated (Table 4), specifically cyclopropyl carbamate analogue **21b** (CB_2_ EC_50_ = 146 nM) as well as cyclobutane-1-carboxamide **21d** and pivalamide **21e** (CB_2_ EC_50_s = 185 and 160 nM, respectively). In this context, the analogous (*R*)-pyranyl compound to **21a** was not characterized; this compound uniquely displayed variable pharmacology between assay runs, specifically loss of activity with subsequent tests. Follow-up studies will be necessary to confirm whether this phenomenon is a result of idiosyncratic instability. Unfortunately, the *des*-hydroxy modification ultimately provided no improvement in the predicted clearance data relative to the hydroxy series.

**Table 4:**
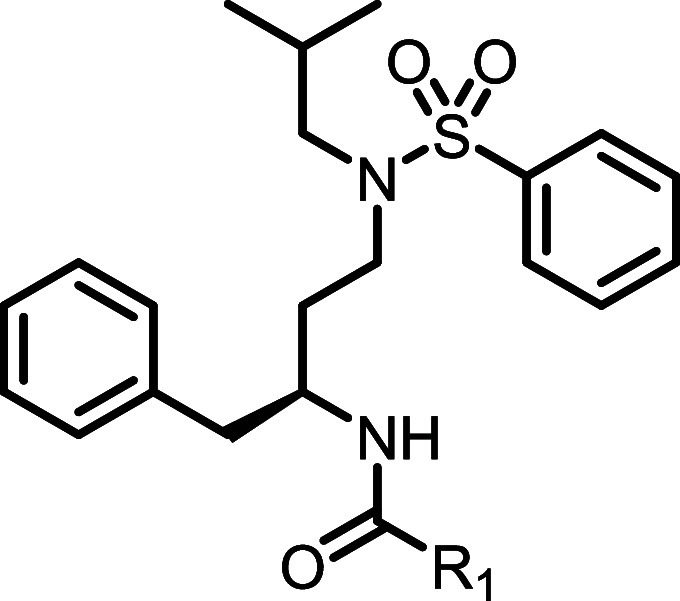

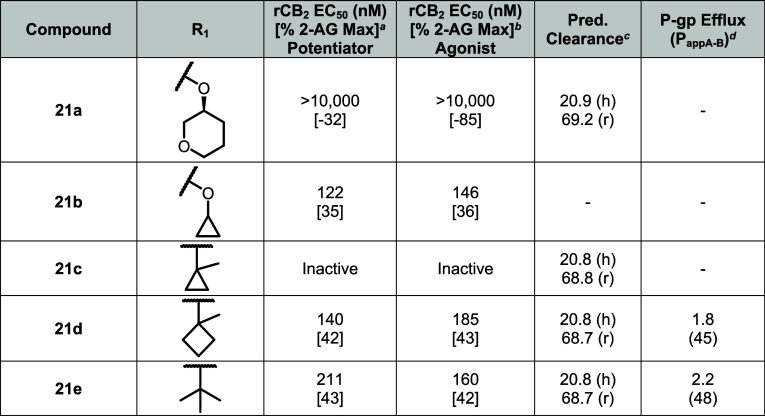
CB_2_ Potency, Predicted Hepatic Clearance, and P-gp Efflux Data for Compounds **21a**–**e** ^a^

a Thallium flux assays in rat CB_2_/GIRK cells run in *a*. the presence and *b*. absence of submaximal 2-AG. EC_50_ values are calculated from the mean pEC_50_ values of at least three independent experiments run in duplicate or triplicate unless otherwise noted. % 2-AG Max represents the maximal compound response as a percentage of 100 μM 2-AG. *c*. Predicted hepatic clearance (microsomes), h = human, r = rat *d*. Efflux ratios for compounds tested in human P-glycoprotein (P-gp) transfected MDCKII-MDR1 cells. P_appA‑B_ is 10^–6^ cm/s. “-” indicates data were not collected.

In order to further understand the *in vitro* and *in vivo* PK profiles of key analogues (and to ascertain the presence of any *in vitro*/*in vivo* disconnect), selected analogues were examined in rat PK PBL experiments (0.2 mg/kg, IV cassette dosing, Table 5).^41^ In general, tested compounds were characterized by moderate to high *in vivo* clearance (CL_p_), short to moderate half-lives (*t*_1/2_ = 0.2–3 h), and a range of *V*_ss_ values (0.47–5.1 L/kg). To understand brain exposure, compound concentrations in plasma and brain were also measured at *t* = 0.25 h in our standard plasma:brain level (PBL) cassette protocol; all tested compounds were found to have moderate–low or negligible brain exposure (comparable to clinical M_1_ PAMs from Merck (MK-7622) and Takeda (TAK-071)). Additionally, all tested compounds were found to have reasonable free fraction values in both human and rat plasma (*f*_u_ = 0.01–0.09; see Table 5).

**Table 5:** CB_2_ and CB_1_ Potency, Plasma Free Fraction, and Rat *In Vivo* PK PBL Data for Selected Compounds^a^

compound	rCB_2_ potency (nM)^a^	rCB_1_ potency (nM)^b^	PPB^c^	CL_p_ (mL/min/kg)^d^	*t*_1/2_ (h)^d^	*V*_ss_ (L/kg)^d^	brain:plasma *K*_p_^d^
**11c**	36.0	>10,000	0.09 (h)	71	2.9	5.1	BLQ brain
			0.08 (r)				
**11h**	161	>10,000	0.09 (h)	54	2.5	3.2	BLQ brain
			0.03 (r)				
**11i**	61.4		0.09 (h)	52	0.22	0.47	BLQ brain
			0.04 (r)				
**11j**	87.8		0.06 (h)	40	0.85	0.72	BLQ brain
			0.03 (r)				
**14b**	148		0.04 (h)	66	1.1	1.8	0.12
			0.03 (r)				
**21d**	185		0.02 (h)	27	1.0	0.57	0.11
			0.01 (r)				
**21e**	160		0.01 (h)	42	0.96	1.18	0.14
			0.02 (r)				

a (a) Thallium flux assays in rat CB_2_/GIRK cells run in the absence of submaximal 2-AG. (b) Thallium flux assays in rat CB_1_/GIRK cells run in the absence of submaximal 2-AG. (c) Plasma protein binding (*f*_u_) via equilibrium dialysis, h = human, r = rat. (d) Rat PK PBL cassette data (male SD rats, 0.2 mg/kg IV dosing; see the Experimental Section and Supporting Information for further experimental details). BLQ = below level of quantitation.

The primary metabolic hotspots of amprenavir itself are well-characterized in the literature^42,43^ and consist primarily of (1) P450-mediated oxidation of the tetrahydrofuran ring, (2) oxidation of the aniline ring, and (3) oxidation of the isobutyl aliphatic chain. Because our next-generation analogues predominantly featured tetrahydrofuran replacements in the context of a *des*-aniline ring system, we examined modifications to the isobutyl side chain in a further attempt to improve the predicted microsomal clearance of the *des*-NH_2_, *des*-hydroxy series. Unfortunately, all examined replacements to the isobutyl group (e.g., methyl, isopropyl) displayed either dramatically attenuated CB_2_ potency and/or did not improve the predicted microsomal clearance. Further efforts will be needed to understand the SAR for the aliphatic side chain, and whether this is a fruitful avenue for the improvement of series PK.

The selectivity of CB_2_ activators relative to the CB_1_ receptor is of critical importance; CB_1_ agonists are often associated with undesirable psychotropic effects that limit their therapeutic utility.^44,45^ Accordingly, we were keen to understand the functional selectivity of additional next-generation compounds relative to the CB_1_ anti-target. Encouragingly, compounds **11c**, **11d**, and **11h** were found to be selective for CB_2_ (see the Supporting Information). As with amprenavir (Figure 2), the rCB_2_ activity of each compound in both the presence and absence of 2-AG was determined, and, for the majority of compounds, found to be comparable, suggesting agonist versus PAM activity (see Tables and the Supporting Information). We also measured human CB_2_ agonist activity in a cell-based arrestin assay and cAMP assay for amprenavir and **11d**. In agreement with the rat CB_2_ GIRK data, amprenavir displayed a human agonist potency of 2.8 μM (50.3% Max) for arrestin, and a human agonist potency of 7.1 μM (54% Max) on cAMP. The more potent rat CB_2_ agonist **11d** also showed improved potency relative to amprenavir on arrestin (human agonist EC_50_ = 59 nM, 111%) and cAMP (human agonist EC_50_ = 100 nM, 109%). Thus, multiple assay readouts for both rat and human CB_2_ confirmed the agonist activity.

To better understand and mechanistically account for the potency trends across the amprenavir series, we coupled extensive fully flexible protein–ligand docking studies with molecular dynamics (MD) simulations to predict the most likely mode of interaction of VU6077967 (**11d**) with CB_2_. Subsequently, we analyzed our SAR trends in the context of the most stable putative docking poses to identify the binding mode most consistent with the observed SAR (e.g., *para* and heteroaromatic substitutions and southern carbamate pendants). We chose VU6077967 (**11d**) as a model compound because of its exceptionally high potency (rCB_2_ EC_50_ = 8.6 nM).

Briefly, we generated homology models of CB_2_ in conformers of all experimentally determined structures of class A GPCRs bound to small-molecule modulators. Similarly, we initialized docking simulations from hundreds of regions of CB_2_ based on the experimentally verified existence of small organic molecules or lipid membrane components found in the template class A GPCRs. For each independent docking simulation, we allowed exchange of the receptor conformer as well as fully flexible side-chain repacking and refinement. This protocol resulted in the identification of five distinct low-energy binding modes across distinct sites in CB_2_ (see the Supporting Information).

To narrow down our five potential binding sites, we performed rCB_2_ radioligand binding assays to determine whether amprenavir (and presumably our analogues) compete with a tritium-labeled synthetic cannabinoid, [^3^H]­CP55,940. We found that amprenavir only weakly inhibits [^3^H]­CP55,940 binding (Figure 3a), suggesting that this chemotype occupies a different binding pocket than 2-AG and the THC-type cannabinoids or only minimally overlaps with this pocket.^35,46^

**Figure 3:**
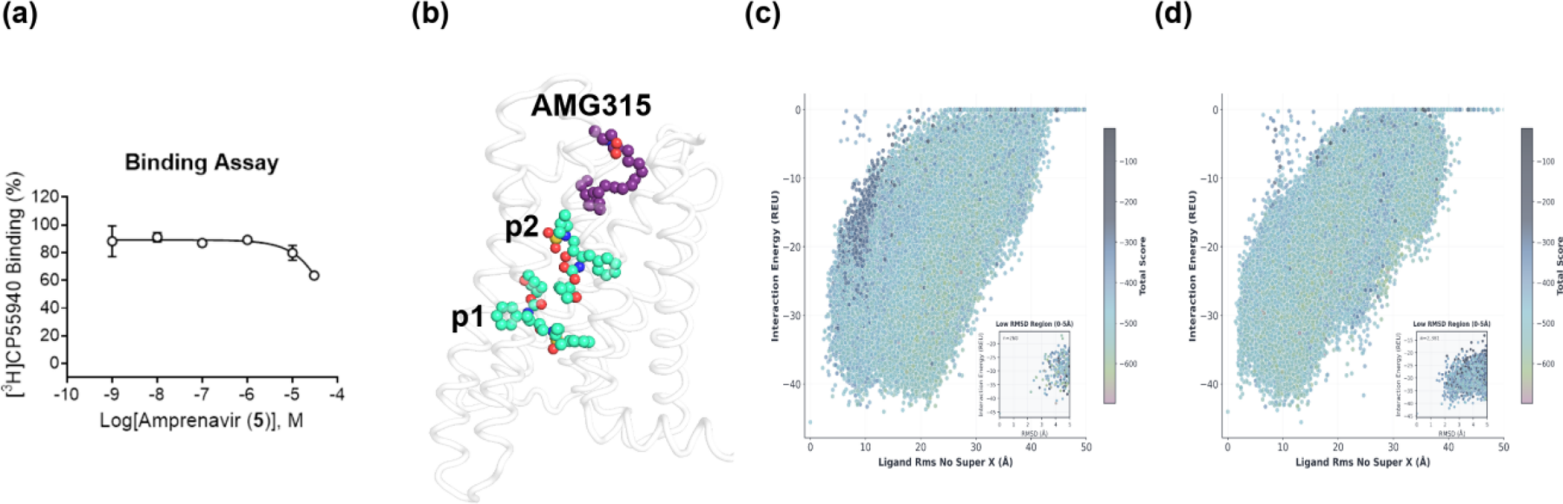
Predicted docking poses of amprenavir series against CB_2_. (a) Amprenavir weakly inhibits [^3^H]­CP55,940 binding under equilibrium conditions. Competition binding concentration–response curves were obtained in the presence of 0.5 nM [^3^H]­CP55,940 using membranes harvested from HEK/GIRK cells expressing rCB_2_. Data represent the mean ± SEM of three independent experiments run in triplicate. Data are plotted as a percentage of specific [^3^H]­CP55,940 binding. Nonspecific binding was determined in the presence of 10 μM CP55,940. (b) The two lowest energy unique binding modes predicted through docking of VU6077967 to CB_2_ (green). Superimposed structure of AMG315 based on the experimental structure of AMG315 with CB_1_ (purple; PDB ID 8GHV). (c) Docking score vs RMSD plot for VU6077967 where RMSDs are computed against pose 1 or (d) pose 2.

Based on these results, we narrowed our putative binding poses from five to two, where the two remaining binding poses were those poses that did not overlap with the experimentally determined binding mode of the synthetic cannabinoid AMG315 with CB_1_ (Figure 3b). Of these two remaining binding poses, pose 2 displayed slightly better convergence in our docking simulations (Figure 3c,d); however, pose 1 overlapped with the corresponding binding mode of a known FFAR3 agonist, AR420626 (see the Supporting Information).

We further interrogated our top two predicted VU6077967 (**11d**) CB_2_ binding poses by performing multiple independent 500 ns replicates of conventional all-atom MD simulations. Our MD simulation results suggested that pose 2 is more stable than pose 1 (Figure 4a–f). Interestingly, pose 2 places the southern carbamate pendant between transmembrane helices 6 and 7 (Figure 5a,b), suggesting a potential mechanism of CB_2_ activation. Furthermore, pose 2 requires mobility of the conserved CWxP W6.48 residue to rotate partially out of the pocket. It has previously been reported in the literature that CB_2_ has higher lability in CWxP W6.48 than the corresponding position in CB_1_. CB_1_ activation requires a well-described ″twin toggle″ mechanism involving coupled translational shifts of W6.48 and F3.36 that disrupt π–π stacking. In contrast, CB_2_ primarily relies on a rotational ∼60–70° χ^2^ dihedral change in W6.48 with minimal F3.36 involvement. Collectively, this has typically been understood to mean that CB_2_ W6.48 conformational transitions occur on faster time scales with lower energetic barriers.^47–49^ In our case, this suggests a plausible mechanism for the intrinsic selectivity of the amprenavir scaffold for CB_2_ over CB_1_ (Figure 5a,b).

**Figure 4:**
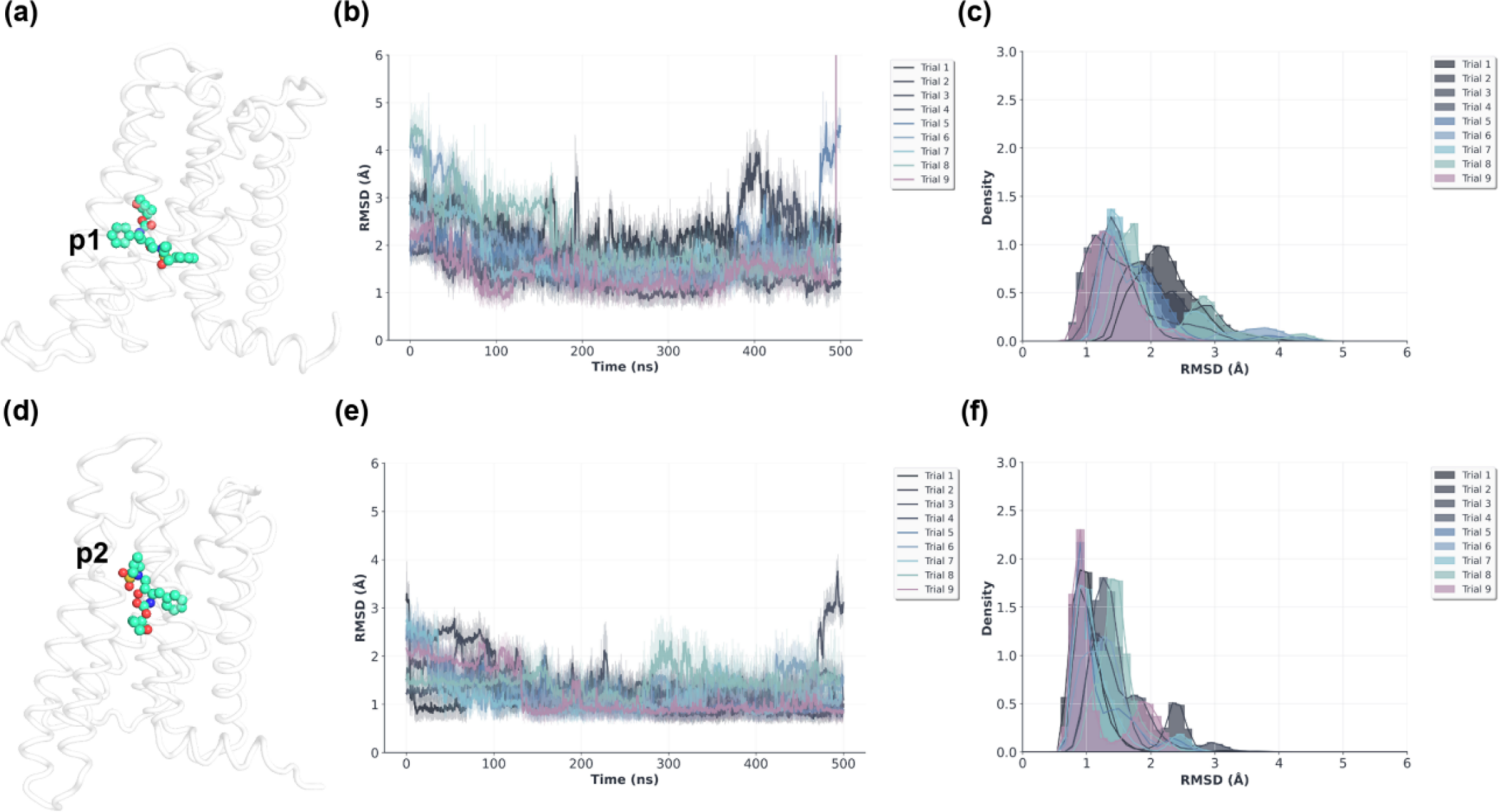
Molecular dynamics simulations of VU6077967 in complex with CB_2_ in each of two putative binding poses. (a) Predicted pose 1, (b) RMSD vs time for pose 1, (c) RMSD distributions for pose 1 simulation trajectories, (d) predicted pose 2, (e) RMSD vs time for pose 2, and (f) RMSD distributions for pose 2 simulation trajectories. In total, nine independent 500 ns trajectories were simulated for each binding pose for a total of 9000 ns of simulation time. All RMSDs are computed with respect to the average ligand pose on a per trajectory basis. All simulation frames were aligned to the first simulation frame based on CB_2_ backbone heavy atom positions prior to ligand coordinate averaging and RMSD calculations.

**Figure 5:**
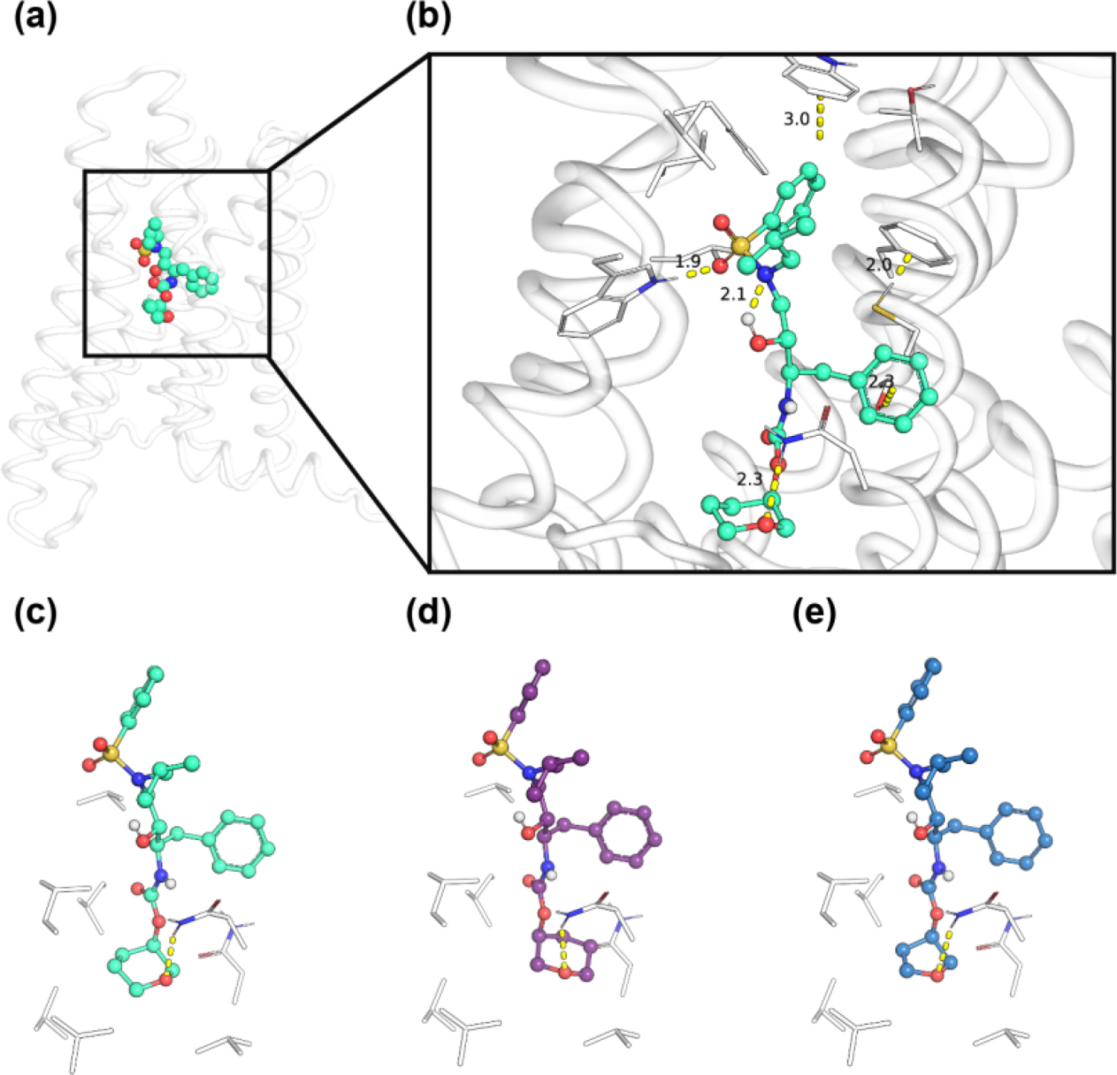
Structural comparison of southern carbamate pendants in predicted VU6077967 (**11d**) CB_2_ binding pose. (a) Zoomed-out structural representation of the docked VU6077967 CB_2_ complex. (b) Zoomed-in structural representation of the docked VU6077967 CB_2_ complex. (c) Focused depiction of VU6077967, (d) VU6077966 (11c), and (e) VU6077969 (11b). CB_2_ transmembrane helix number indicated with lowercase roman numerals.

Finally, the sensitivity of the amprenavir scaffold to modifications at the southern carbamate pendant is consistent with predicted interactions with a helix 7 asparagine and neighboring hydrophobic residues (Figure 5c–e). The (*R*)-pyranyl of VU6077967 (**11d**) is oriented to be a strong hydrogen bond acceptor for the helix 7 asparagine while simultaneously filling the hydrophobic pocket. Substitution with (*S*)-pyranyl necessitates poorer steric interactions within the pocket to satisfy a hydrogen bond with the same asparagine. Similarly, (*R*)-furanyl displaces the acceptor oxygen atom downward and does not fill the pocket as much as the pyranyl, likely resulting in enhanced fluctuations and worse specific contacts. Altogether, our modeling provides a potential basis for selectivity of CB_2_ over CB_1_ and additionally suggests a mechanistic basis for allosteric agonism of CB_2_ by amprenavir-based scaffolds that is consistent with our SAR and pharmacological data.

## Conclusions

In conclusion, we have identified the HIV protease inhibitor amprenavir as an activator for the CB_2_ receptor in a thallium flux rat CB_2_/GIRK assay. SAR on this scaffold ultimately led to the identification of next-generation analogues with excellent CB_2_ potency (EC_50_s < 10 nM), and low predicted P-gp efflux compared to amprenavir, highlighting the tractability of this scaffold for the eventual development of brain-penetrant analogs. Unfortunately, no chemical modifications were found to improve the predicted hepatic microsomal clearance of this scaffold, and a further understanding of the metabolic hotspot(s) of our novel analogues will be necessary for continued scaffold development. Nevertheless, our modeling results provide a rationale for selectivity over CB_1_ and present actionable mechanistic hypotheses for further scaffold development and compound prioritization; however, future targeted mutagenesis experiments or cryo-EM studies will be required to validate the predicted binding mode. As a template scaffold for further drug development and as an additional tool to understand the binding topography of the CB_2_ receptor, it is our hope that the amprenavir chemotype will be of further use to medicinal chemists working in the cannabinoid space. Specifically, the emergence of new structural information on the CB_2_ receptor,^50–52^ along with an increased understanding of druggable pockets, may enable the rational design of next-generation amprenavir-based CB_2_ activators (and raises the intriguing possibility that other existing protease inhibitors may already have CB_2_ activity as part of their broader pharmacology).

## Experimental Section

### General Experimental Information

All reactions were carried out employing standard chemical techniques. Solvents used for reactions and extraction were of ACS grade, and HPLC-grade solvents were used for purification. All reagents were purchased from commercial sources and were used without further purification. Amprenavir (CAS 161814-49-9) was purchased from Aurum Pharmatech. All NMR spectra were recorded on a 400 MHz Bruker AV-400 instrument. ^1^H chemical shifts are reported as δ values in ppm relative to the residual solvent peak (CDCl_3_ = 7.26, DMSO-*d*_6_ = 2.50, CD_3_OD = 3.31). ^13^C chemical shifts are reported as δ values in ppm relative to the residual solvent peak (CDCl_3_ = 77.16, DMSO-*d*_6_ = 39.52, CD_3_OD = 49.00). Data are reported as follows: chemical shift, multiplicity (br = broad, s = singlet, d = doublet, t = triplet, q = quartet, p = pentet, dd = doublet of doublets, ddd = doublet of doublet of doublets, td = triplet of doublets, m = multiplet), coupling constant, and integration. LC-MS data were obtained on a Waters QDa (Performance) SQ MS with an ESI source. MS parameters were as follows: cone voltage: 15 V, capillary voltage: 0.8 kV, probe temperature: 600 °C. Samples were introduced via an ACQUITY I-Class PLUS UPLC composed of a BSM, FLSM, CH-A, and PDA. UV absorption was generally observed at 215 and 254 nm; 4 nm bandwidth. Column: Phenomenex EVO C18, 1.0 × 50 mm, 1.7 μm. Column temperature: 55 °C. Flow rate: 0.4 mL/min. Default gradient: 5 to 95% CH_3_CN (0.05% TFA) in water (0.05% TFA) over 1.4 min, hold at 95% CH_3_CN for 0.1 min. High-resolution mass spectra were obtained on an Agilent 6540 UHD Q-TOF with an ESI source. MS parameters were as follows: fragmentor: 150, capillary voltage: 3500 V, nebulizer pressure: 60 psig, drying gas flow: 13 L/min, drying gas temperature: 275 °C. Samples were introduced via an Agilent 1290 UHPLC composed of a G4220A binary pump, G4226A ALS, G1316C TCC, and G4212A DAD with a ULD flow cell. UV absorption was observed at 215 and 254 nm with a 4 nm bandwidth. Column: Agilent ZORBAX Extend-C18, 1.8 μm, 2.1 × 50 mm. Gradient conditions: 5 to 95% MeCN in water (0.1% formic acid) over 1 min, hold at 95% MeCN for 0.1 min, 0.5 mL/min, 40 °C. RP-HPLC purifications were performed on a Gilson preparative reversed-phase HPLC system composed of a 333 aqueous pump with a solvent-selection valve, a 334 organic pump, a GX-271 or GX-281 liquid hander, two column switching valves, and a 155 UV detector. UV wavelength for fraction collection was user-defined, with absorbance generally monitored at 220 nm. Column: Phenomenex Axia-packed Gemini C18, 30 × 50 mm or 30 × 100 mm, 5 μm. Mobile phase: MeCN in H_2_O (0.1% TFA) or MeCN in H_2_O (0.05% v/v NH_4_OH). Gradient conditions: 0.75 min equilibration, followed by user-defined gradient (starting organic percentage, ending organic percentage, duration), hold at 95% MeCN for 1 min, 50 mL/min, 23 °C. All tested compounds were ≥95% purity as assessed by LC-MS and ^1^H NMR analysis. Automated flash column chromatography was performed on a Biotage Isolera 1 or a Teledyne ISCO CombiFlash system.

### Synthesis of Compounds **11a** and **14a**–**i** (Scheme 1)

#### Benzyl ((2*R*,3*S*)-3-((*tert*-Butoxycarbonyl)­amino)-2-hydroxy-4-phenylbutyl)­(isobutyl)­carbamate (**7**)

To a solution of *tert*-butyl ((2*S*,3*R*)-3-hydroxy-4-(isobutylamino)-1-phenylbutan-2-yl)­carbamate (100 mg, 0.3 mmol, 1 equiv) in THF (0.8 mL) was added a solution of potassium carbonate (83.3 mg, 0.59 mmol, 2 equiv) in water (0.5 mL), and the resulting mixture was cooled to 0 °C. Benzyl chloroformate (0.05 mL, 0.36 mmol, 1.2 equiv) dissolved in THF (0.4 mL) was added dropwise to the above mixture, and the reaction was slowly warmed to r.t. and stirred for 3 h. Upon completion, the reaction mixture was diluted with ethyl acetate and the layers were separated. Organics were washed sequentially by saturated NaHCO_3_ solution and brine, dried over Na_2_SO_4_, filtered, and concentrated. Crude residue was purified by column chromatography (0–60% EtOAc in hexanes) to give the title compound as a clear gel (89 mg, 64%). ^1^H NMR (400 MHz, DMSO-*d*_6_) δ 7.41 – 7.12 (m, 10H), 6.65 (t, *J* = 9.5 Hz, 1H), 5.15 – 4.92 (m, 3H), 3.70 – 3.61 (m, 1H), 3.56 – 3.43 (m, 2H), 3.26 – 2.90 (m, 4H), 1.98 – 1.84 (m, 1H), 1.26 (s, 9H), 0.89 – 0.75 (m, 6H). ^13^C NMR (101 MHz, DMSO-*d*_6_) δ 155.8, 155.4, 139.8, 139.6, 137.4, 137.2, 129.2, 129.2, 128.5, 128.4, 128.0, 128.0, 127.8, 127.6, 127.5, 127.2, 125.7, 77.5, 71.9, 71.4, 66.1, 65.9, 55.4, 55.3, 54.7, 54.5, 50.8, 50.2, 35.7, 28.2, 27.8, 26.9, 26.3, 20.0, 19.9. HRMS (TOF, ES+), C_27_H_39_N_2_O_5_ [M + H]^+^ calc. mass 471.2853, found 471.2858.

#### Benzyl ((2*R*,3*S*)-3-Amino-2-hydroxy-4-phenylbutyl)­(isobutyl)­carbamate (**8**)

To a solution of **7** (77 mg, 0.16 mmol, 1 equiv) in 1,4-dioxane (0.4 mL) was added hydrochloric acid (0.41 mL, 1.64 mmol, 10 equiv) (4 M in 1,4-dioxane), and the reaction mixture was stirred at r.t. for 2 h. Upon completion, the reaction mixture was concentrated under vacuum to give the title compound as a tan glass, which was carried forward without purification (assuming theoretical yield). ^1^H NMR (400 MHz, MeOD) δ 7.46 – 7.13 (m, 10H), 5.21 – 5.05 (m, 2H), 4.13 – 4.03 (m, 1H), 3.78 – 3.42 (m, 3H), 3.24 – 3.04 (m, 3H), 2.91 – 2.80 (m, 1H), 2.04 – 1.88 (m, 1H), 0.87 (d, *J* = 6.8 Hz, 3H), 0.84 (d, *J* = 6.4 Hz, 3H). ^13^C NMR (101 MHz, MeOD) δ 158.7, 158.0, 138.1, 138.0, 137.3, 137.1, 130.5, 130.3, 130.1, 129.6, 129.2, 129.1, 129.0, 128.5, 73.5, 72.4, 70.2, 70.0, 68.5, 68.4, 62.2, 57.1, 57.0, 56.7, 56.6, 51.2, 34.3, 34.0, 28.6, 28.0, 20.4, 20.3. HRMS (TOF, ES+), C_22_H_31_ClN_2_O_3_ [M + H]^+^ calc. mass 371.2329, found 371.2335.

#### Benzyl ((2*R*,3*S*)-2-Hydroxy-4-phenyl-3-(((((*S*)-tetrahydrofuran-3-yl)­oxy)­carbonyl)­amino)­butyl)­(isobutyl)­carbamate (**9a**)

To a suspension of *N,N*′-disuccinimidyl carbonate (15.1 mg, 0.06 mmol, 1.5 equiv) in MeCN (0.5 mL) were added (*S*)-tetrahydrofuran-3-ol (10.4 mg, 0.12 mmol, 3 equiv) and pyridine (0.15 mL), and the mixture was stirred at r.t. for 1 h before the sequential addition of **8** (16 mg, 0.04 mmol, 1 equiv) in MeCN (0.5 mL) and triethylamine (0.01 mL, 0.05 mmol, 1.2 equiv). The reaction mixture was stirred at r.t. for 1 h. Upon completion, the reaction mixture was diluted with water and extracted with DCM. Combined organic extracts were filtered through a hydrophobic phase separator and concentrated. Crude residue was purified by column chromatography (0–100% EtOAc in hexanes) to give the title compound as a clear glass (7.2 mg, 38%). ^1^H NMR (400 MHz, DMSO-*d*_6_) δ 7.41 – 7.28 (m, 5H), 7.26 – 7.06 (m, 6H), 5.14 – 5.00 (m, 3H), 4.97 – 4.81 (m, 1H), 3.76 – 3.43 (m, 6H), 3.29 – 2.90 (m, 4H), 2.58 – 2.51 (m, 1H), 2.10 – 1.69 (m, 3H), 0.84 – 0.76 (m, 6H). ^13^C NMR (101 MHz, DMSO-*d*_6_) δ 155.7, 155.6, 139.5, 139.4, 137.3, 137.1, 129.1, 129.1, 128.4, 128.3, 127.9, 127.7, 127.6, 127.5, 127.1, 125.7, 74.1, 72.6, 71.8, 71.3, 66.1, 66.1, 65.8, 55.8, 54.7, 54.5, 50.6, 50.0, 35.6, 35.5, 32.2, 26.8, 26.3, 19.9, 19.8. HRMS (TOF, ES+), C_27_H_37_N_2_O_6_ [M + H]^+^ calc. mass 485.2646, found 485.2644.

#### Benzyl ((2*R*,3*S*)-2-Hydroxy-4-phenyl-3-(((((*R*)-tetrahydro-2*H*-pyran-3-yl)­oxy)­carbonyl)­amino)­butyl)­(isobutyl)­carbamate (**9b**)

To a suspension of *N*,*N*′-disuccinimidyl carbonate (38.7 mg, 0.15 mmol, 1.5 equiv) in MeCN (0.5 mL) were added (*R*)-tetrahydro-2*H*-pyran-3-ol (30.9 mg, 0.3 mmol, 3 equiv) and pyridine (0.15 mL), and the mixture was stirred at r.t. for 1 h before the sequential addition of 8 (41 mg, 0.1 mmol, 1 equiv) in MeCN (0.5 mL) and triethylamine (0.02 mL, 0.12 mmol, 1.2 equiv). The reaction mixture was stirred at r.t. for 1 h. Upon completion, the reaction mixture was diluted with water and extracted with DCM. Combined organic extracts were filtered through a hydrophobic phase separator and concentrated. The crude residue was purified by column chromatography (0–100% EtOAc in hexanes) to give the title compound as a clear glass (32 mg, 64%). ^1^H NMR (400 MHz, DMSO-*d*_6_) δ 7.40 – 7.27 (m, 5H), 7.25 – 7.11 (m, 5H), 7.06 (t, *J* = 7.6 Hz, 1H), 5.16 – 4.98 (m, 3H), 4.37 – 4.22 (m, 1H), 3.72 – 3.43 (m, 6H), 3.24 – 2.88 (m, 4H), 2.58 – 2.45 (m, 1H), 1.97 – 1.84 (m, 1H), 1.72 – 1.59 (m, 2H), 1.48 – 1.31 (m, 2H), 0.85 – 0.75 (m, 6H). ^13^C NMR (101 MHz, DMSO-*d*_6_) δ 155.8, 155.3, 139.6, 139.5, 137.3, 137.1, 129.2, 129.1, 128.4, 128.3, 127.9, 127.7, 127.5, 127.5, 127.1, 125.7, 71.8, 71.3, 69.2, 68.9, 67.2, 66.8, 66.1, 65.8, 55.9, 54.7, 54.5, 50.7, 50.0, 35.7, 35.6, 27.8, 26.9, 26.3, 23.0, 22.6, 19.9, 19.8. HRMS (TOF, ES+), C_28_H_39_N_2_O_6_ [M + H]^+^ calc. mass 499.2803, found 499.2803.

#### Benzyl ((2*R*,3*S*)-2-Hydroxy-4-phenyl-3-(((((*S*)-tetrahydrofuran-3-yl)­oxy)­carbonyl)­amino)­butyl)­(isobutyl)­carbamate (**10a**)

To a solution of **9a** (25 mg, 0.05 mmol, 1 equiv) in MeOH (1 mL) was added palladium hydroxide on activated carbon (20 wt %) (3.6 mg, 0.01 mmol, 0.1 equiv), and the reaction vessel was evacuated and then placed under an H_2_ atmosphere and stirred at r.t. overnight. Upon completion, the reaction mixture was diluted with MeOH, and solids were removed by syringe filtration. Solvents were concentrated to give the title compound as a white solid (17.8 mg, 98%). ^1^H NMR (400 MHz, DMSO-*d*_6_) δ 7.20 – 7.14 (m, 2H), 7.13 – 7.03 (m, 4H), 4.92 – 4.85 (m, 1H), 3.69 – 3.46 (m, 4H), 3.37 (td, *J* = 7.4, 3.4 Hz, 1H), 3.33 – 3.17 (m, 1H), 2.93 (dd, *J* = 13.8, 3.6 Hz, 1H), 2.55 – 2.37 (m, 3H), 2.24 (d, *J* = 6.7 Hz, 2H), 2.04 – 1.92 (m, 1H), 1.76 – 1.66 (m, 1H), 1.66 – 1.50 (m, *J* = 6.7 Hz, 1H), 0.80 (d, *J* = 1.1 Hz, 3H), 0.79 (d, *J* = 1.1 Hz, 3H). ^13^C NMR (101 MHz, DMSO-*d*_6_) δ 155.6, 154.9, 139.7, 139.6, 129.2, 129.1, 128.0, 127.9, 125.9, 125.7, 76.7, 74.2, 74.0, 72.6, 72.3, 71.6, 71.5, 66.1, 66.0, 61.9, 57.6, 56.8, 55.9, 52.7, 37.0, 36.2, 32.3, 27.9, 20.6, 20.6. HRMS (TOF, ES+), C_19_H_31_N_2_O_4_ [M + H]^+^ calc. mass 351.2278, found 351.2278.

#### (*R*)-Tetrahydro-2*H*-pyran-3-yl ((2*S*,3*R*)-3-Hydroxy-4-(isobutylamino)-1-phenylbutan-2-yl)­carbamate) (**10b**)

To a solution of **9b** (32 mg, 0.06 mmol, 1 equiv) in MeOH (1 mL) was added palladium hydroxide on activated carbon (20 wt %) (4.5 mg, 0.01 mmol, 0.1 equiv), and the reaction vessel was evacuated and then placed under an H_2_ atmosphere and stirred at r.t. overnight. Upon completion, the reaction mixture was diluted with MeOH, and solids were removed by syringe filtration. Solvents were concentrated to give the title compound as a white solid (22 mg, 94%). ^1^H NMR (400 MHz, MeOD) δ 7.28 – 7.20 (m, 4H), 7.19 – 7.13 (m, 1H), 4.45 – 4.32 (m, 1H), 3.79 – 3.45 (m, 6H), 3.12 (dd, *J* = 13.8, 3.8 Hz, 1H), 2.82 – 2.71 (m, 1H), 2.66 – 2.54 (m, 2H), 2.47 (dd, *J* = 11.7, 6.8 Hz, 1H), 2.38 (dd, *J* = 11.7, 6.9 Hz, 1H), 1.94 – 1.68 (m, 3H), 1.57 – 1.39 (m, 2H), 0.94 (d, *J* = 6.7 Hz, 6H). ^13^C NMR (101 MHz, MeOD) δ 158.0, 140.2, 130.5, 130.4, 129.3, 129.2, 129.2, 127.1, 73.2, 73.0, 70.9, 70.9, 70.5, 69.9, 69.5, 69.5, 68.8, 68.7, 58.7, 57.8, 57.7, 53.6, 53.5, 38.6, 38.0, 29.4, 29.1, 29.1, 24.3, 24.2, 23.8, 21.0, 20.9. HRMS (TOF, ES+), C_20_H_33_N_2_O_4_ [M + H]^+^ calc. mass 365.2435, found 365.2436.

#### (*S*)-Tetrahydrofuran-3-yl ((2*S*,3*R*)-3-Hydroxy-4-(*N*-isobutylphenylsulfonamido)-1-phenylbutan-2-yl)­carbamate (**11a**)

To a solution of **10a** (12 mg, 0.034 mmol, 1 equiv) in DCM (0.8 mL) was added a solution of NaHCO_3_ (5.7 mg, 0.069 mmol, 2 equiv) in H_2_O (0.25 mL). The resulting reaction mixture was cooled to 0 °C, after which time benzenesulfonyl chloride (4.8 μL, 0.038 mmol, 1.1 equiv) was added. The resulting reaction mixture was stirred at r.t. overnight, after which time sat. NaHCO_3_ solution was added, and the aqueous layer was extracted with DCM. Combined organic extracts were filtered through a hydrophobic phase separator and concentrated. The crude residue was purified by RP-HPLC (25–65% MeCN in 0.1% aqueous TFA solution over 5 min). Fractions containing the product were basified with sat. NaHCO_3_ solution and extracted with EtOAc. Combined organic extracts were filtered through a hydrophobic phase separator and concentrated to afford the title compound as a clear gel (12.1 mg, 72%). ^1^H NMR (400 MHz, DMSO-*d*_6_) δ 7.81 – 7.76 (m, 2H), 7.69 – 7.64 (m, 1H), 7.63 – 7.56 (m, 2H), 7.27 – 7.10 (m, 6H), 5.03 (d, *J* = 6.7 Hz, 1H), 4.97 – 4.91 (m, 1H), 3.76 – 3.49 (m, 5H), 3.40 – 3.31 (m, 2H), 3.08 – 2.96 (m, 2H), 2.91 – 2.77 (m, 2H), 2.11 – 1.91 (m, 2H), 1.82 – 1.73 (m, 1H), 0.84 (d, *J* = 6.5 Hz, 3H), 0.78 (d, *J* = 6.6 Hz, 3H). ^13^C NMR (101 MHz, DMSO-*d*_6_) δ 155.6, 139.5, 139.2, 132.6, 129.2, 129.1, 127.9, 127.0, 125.8, 74.1, 72.6, 72.1, 66.1, 56.4, 55.7, 52.1, 35.3, 32.3, 26.1, 19.9, 19.9. HRMS (TOF, ES+), C_25_H_35_N_2_O_6_S [M + H]^+^ calc. mass 491.2210, found 491.2210.

#### (*R*)-Tetrahydro-2*H*-pyran-3-yl ((2*S*,3*R*)-4-((4-Fluoro-*N*-isobutylphenyl)­sulfonamido)-3-hydroxy-1-phenylbutan-2-yl)­carbamate (**14a**)

The procedure for **11a** with **10b** (12 mg, 0.03 mmol, 1 equiv) and 4-fluorobenzenesulfonyl chloride (7 mg, 0.04 mmol, 1.1 equiv) to give the title compound as a white solid after purification by RP-HPLC (25–65% MeCN in 0.1% aqueous TFA solution over 10 min) (8.5 mg, 49%) was followed. ^1^H NMR (400 MHz, DMSO-*d*_6_) δ 7.89 – 7.82 (m, 2H), 7.46 – 7.38 (m, 2H), 7.27 – 7.06 (m, 6H), 5.02 (d, *J* = 6.5 Hz, 1H), 4.31 (m, 1H), 3.63 – 3.43 (m, 5H), 3.38 – 3.28 (m, 2H), 3.09 – 2.78 (m, 4H), 2.02 – 1.89 (m, 1H), 1.72 – 1.59 (m, 2H), 1.45 – 1.33 (m, 2H), 0.84 (d, *J* = 6.5 Hz, 3H), 0.79 (d, *J* = 6.6 Hz, 3H). ^13^C NMR (101 MHz, DMSO-*d*_6_) δ 164.2 (d, *J* = 251.5 Hz), 155.3, 139.4, 135.8 (d, *J* = 3.0 Hz), 130.1 (d, *J* = 9.1 Hz), 129.2, 127.9, 125.8, 116.3 (d, *J* = 22.2 Hz), 71.8, 69.2, 67.3, 66.8, 56.0, 55.8, 51.8, 35.4, 27.7, 26.0, 22.5, 19.9, 19.8. HRMS (TOF, ES+), C_26_H_36_FN_2_O_6_S [M + H]^+^ calc. mass 523.2273, found 523.2270.

#### (*R*)-Tetrahydro-2*H*-pyran-3-yl ((2*S*,3*R*)-4-((4-Chloro-*N*-isobutylphenyl)­sulfonamido)-3-hydroxy-1-phenylbutan-2-yl)­carbamate (**14b**)

The procedure for **11a** with **10b** (12 mg, 0.03 mmol, 1 equiv) and 4-chlorobenzenesulfonyl chloride (7.6 mg, 0.04 mmol, 1.1 equiv) to give the title compound as a white solid after purification by RP-HPLC (25–65% MeCN in 0.1% aqueous TFA solution over 10 min) (9.9 mg, 56%) was followed. ^1^H NMR (400 MHz, DMSO-*d*_6_) δ 7.83 – 7.77 (m, 2H), 7.68 – 7.63 (m, 2H), 7.27 – 7.11 (m, 6H), 5.06 (d, *J* = 6.5 Hz, 1H), 4.35 – 4.21 (m, 1H), 3.63 – 3.42 (m, 5H), 3.38 – 3.27 (m, 2H), 3.10 – 2.78 (m, 4H), 2.02 – 1.89 (m, 1H), 1.71 – 1.60 (m, 2H), 1.46 – 1.31 (m, 2H), 0.83 (d, *J* = 6.5 Hz, 3H), 0.79 (d, *J* = 6.6 Hz, 3H). ^13^C NMR (101 MHz, DMSO-*d*_6_) δ 155.4, 139.5, 138.3, 137.5, 129.4, 129.2, 129.1, 128.0, 125.8, 71.7, 69.2, 67.3, 66.9, 56.0, 55.9, 51.8, 35.5, 27.8, 26.0, 22.6, 20.0, 19.9. HRMS (TOF, ES+), C_26_H_36_ClN_2_O_6_S [M + H]^+^ calc. mass 539.1977, found 539.1976.

#### (*R*)-Tetrahydro-2*H*-pyran-3-yl ((2*S*,3*R*)-3-Hydroxy-4-((*N*-isobutyl-4-methylphenyl)­sulfonamido)-1-phenylbutan-2-yl)­carbamate (**14c**)

The procedure for **11a** with **10b** (12 mg, 0.03 mmol, 1 equiv) and 4-toluenesulfonyl chloride (6.9 mg, 0.04 mmol, 1.1 equiv) to give the title compound as a white solid after purification by RP-HPLC (25–65% MeCN in 0.1% aqueous TFA solution over 10 min) (8.4 mg, 49%) was followed. ^1^H NMR (400 MHz, DMSO-*d*_6_) δ 7.69 – 7.64 (m, 2H), 7.38 (d, *J* = 8.1 Hz, 2H), 7.26 – 7.11 (m, 6H), 5.05 (d, *J* = 6.6 Hz, 1H), 4.34 – 4.20 (m, 1H), 3.65 – 3.45 (m, 5H), 3.38 – 3.26 (m, 2H), 3.04 – 2.94 (m, 2H), 2.89 – 2.70 (m, 2H), 2.38 (s, 3H), 2.02 – 1.90 (m, 1H), 1.73 – 1.60 (m, 2H), 1.43 – 1.30 (m, 2H), 0.84 (d, *J* = 6.5 Hz, 3H), 0.78 (d, *J* = 6.6 Hz, 3H). ^13^C NMR (101 MHz, DMSO-*d*_6_) δ 155.4, 143.0, 139.6, 136.1, 129.7, 129.3, 127.9, 127.2, 125.8, 72.2, 69.3, 67.3, 66.9, 56.7, 55.9, 52.4, 35.4, 27.8, 26.3, 22.6, 21.1, 20.0, 20.0. HRMS (TOF, ES+), C_27_H_39_N_2_O_6_S [M + H]^+^ calc. mass 519.2523, found 519.2530.

#### (*R*)-Tetrahydro-2*H*-pyran-3-yl ((2*S*,3*R*)-3-Hydroxy-4-(*N*-isobutylcyclopropanesulfonamido)-1-phenylbutan-2-yl)­carbamate (**14d**)

The procedure for **11a** with **10b** (10 mg, 0.03 mmol, 1 equiv) and cyclopropanesulfonyl chloride (4.6 mg, 0.03 mmol, 1.1 equiv) to give the title compound as a white solid after purification by RP-HPLC (20–80% MeCN in 0.05% aqueous NH_4_OH solution over 5 min) (4.4 mg, 34%) was followed. ^1^H NMR (400 MHz, DMSO-*d*_6_) δ 7.27 – 7.11 (m, 6H), 5.18 (d, *J* = 6.9 Hz, 1H), 4.34 – 4.22 (m, 1H), 3.71 – 3.45 (m, 5H), 3.41 – 3.29 (m, 2H), 3.15 – 2.94 (m, 4H), 2.73 – 2.64 (m, 1H), 1.98 – 1.86 (m, 1H), 1.71 – 1.59 (m, 2H), 1.44 – 1.30 (m, 2H), 0.98 – 0.89 (m, 4H), 0.86 (d, *J* = 2.3 Hz, 3H), 0.84 (d, *J* = 2.4 Hz, 3H). ^13^C NMR (101 MHz, DMSO-*d*_6_) δ 155.4, 139.6, 129.2, 128.0, 125.8, 71.6, 69.2, 67.3, 66.9, 55.9, 54.9, 50.9, 35.6, 28.5, 27.8, 26.0, 22.6, 20.0, 19.8, 4.6, 4.4. HRMS (TOF, ES+), C_23_H_37_N_2_O_6_S [M + Na]^+^ calc. mass 491.2192, found 491.2189.

#### (*R*)-Tetrahydro-2*H*-pyran-3-yl ((2*S*,3*R*)-3-Hydroxy-4-((*N*-isobutyl-2-methyloxazole)-5-sulfonamido)-1-phenylbutan-2-yl)­carbamate (**14e**)

The procedure for **11a** with **10b** (11 mg, 0.03 mmol, 1 equiv) and 2-methyloxazole-5-sulfonyl chloride (6.6 mg, 0.04 mmol, 1.1 equiv) to give the title compound as a white solid after purification by RP-HPLC (20–80% MeCN in 0.05% aqueous NH_4_OH solution over 5 min) (5.5 mg, 36%) was followed. ^1^H NMR (400 MHz, DMSO-*d*_6_) δ 7.66 – 7.62 (m, 1H), 7.28 – 7.13 (m, 6H), 5.16 (d, *J* = 6.8 Hz, 1H), 4.36 – 4.24 (m, 1H), 3.67 – 3.56 (m, 2H), 3.55 – 3.46 (m, 3H), 3.45 – 3.31 (m, 2H), 3.19 – 3.10 (m, 1H), 3.08 – 2.95 (m, 3H), 2.50 (s, 3H), 2.05 – 1.93 (m, 1H), 1.71 – 1.61 (m, 2H), 1.47 – 1.32 (m, 2H), 0.83 (d, *J* = 6.5 Hz, 3H), 0.81 (d, *J* = 6.5 Hz, 3H). ^13^C NMR (101 MHz, DMSO-*d*_6_) δ 164.3, 155.4, 146.3, 139.4, 131.5, 129.2, 128.0, 125.8, 71.4, 69.2, 67.3, 66.9, 55.9, 55.7, 51.5, 35.6, 27.8, 26.0, 22.6, 19.8, 19.7, 13.9. HRMS (TOF, ES+), C_24_H_36_N_3_O_7_S [M + H]^+^ calc. mass 510.2268, found 510.2272.

#### (*R*)-Tetrahydro-2*H*-pyran-3-yl ((2*S*,3*R*)-3-Hydroxy-4-((*N*-isobutyl-2-methylthiazole)-5-sulfonamido)-1-phenylbutan-2-yl)­carbamate (**14f**)

The procedure for **11a** with **10b** (10 mg, 0.03 mmol, 1 equiv) and 2-methylthiazole-5-sulfonyl chloride (6 mg, 0.03 mmol, 1.1 equiv) to give the title compound as a tan glass after purification by column chromatography (0–80% EtOAc in hexanes) (14 mg, 65%) was followed. ^1^H NMR (400 MHz, DMSO-*d*_6_) δ 8.08 (s, 1H), 7.28 – 7.09 (m, 6H), 5.12 (d, *J* = 6.7 Hz, 1H), 4.37 – 4.22 (m, 1H), 3.69 – 3.57 (m, 2H), 3.57 – 3.43 (m, 3H), 3.40 – 3.35 (m, 2H), 3.09 – 2.79 (m, 4H), 2.72 (s, 3H), 2.08 – 1.95 (m, 1H), 1.72 – 1.60 (m, 2H), 1.48 – 1.31 (m, 2H), 0.87 (d, *J* = 6.5 Hz, 3H), 0.83 (d, *J* = 6.6 Hz, 3H). ^13^C NMR (101 MHz, DMSO-*d*_6_) δ 171.6, 155.3, 145.8, 139.4, 134.5, 129.1, 129.1, 127.9, 127.9, 125.7, 71.8, 71.8, 69.2, 67.3, 66.8, 66.8, 56.7, 56.5, 55.9, 54.9, 52.7, 35.5, 28.1, 27.8, 26.3, 26.2, 22.9, 22.6, 19.9, 19.8, 19.2. HRMS (TOF, ES+), C_24_H_36_N_3_O_6_S_2_ [M + H]^+^ calc. mass 526.2040, found 526.2041.

#### (*R*)-Tetrahydro-2*H*-pyran-3-yl­((2*S*,3*R*)-3-hydroxy-4-((*N*-isobutyl-3-methylisothiazole)-5-sulfonamido)-1-phenylbutan-2-yl)­carbamate (**14g**)

The procedure for **11a** with **10b** (11 mg, 0.03 mmol, 1 equiv) and 3-methylisothiazole-5-sulfonyl chloride (7.2 mg, 0.04 mmol, 1.2 equiv) to give the title compound as a white solid after purification by RP-HPLC (20–80% MeCN in 0.05% aqueous NH_4_OH solution over 10 min) (6.5 mg, 41%) was followed. ^1^H NMR (400 MHz, DMSO-*d*_6_) δ 7.71 (s, 1H), 7.27 – 7.12 (m, 6H), 5.20 (d, *J* = 6.8 Hz, 1H), 4.35 – 4.24 (m, 1H), 3.66 – 3.57 (m, 2H), 3.56 – 3.43 (m, 3H), 3.42 – 3.29 (m, 2H), 3.13 – 3.05 (m, 1H), 3.02 – 2.93 (m, 2H), 2.92 – 2.84 (m, 1H), 2.48 (s, 3H), 2.08 – 1.97 (m, 1H), 1.71 – 1.60 (m, 2H), 1.44 – 1.31 (m, 2H), 0.87 (d, *J* = 6.6 Hz, 3H), 0.84 (d, *J* = 6.6 Hz, 3H). ^13^C NMR (101 MHz, DMSO-*d*_6_) δ 168.2, 162.8, 155.4, 139.4, 129.2, 128.0, 126.8, 125.9, 71.7, 69.3, 67.3, 66.9, 56.5, 55.9, 52.6, 35.6, 27.8, 26.2, 22.6, 19.9, 19.8, 18.9. HRMS (TOF, ES+), C_24_H_36_N_3_O_6_S_2_ [M + H]^+^ calc. mass 526.2040, found 526.2035.

#### (*R*)-Tetrahydro-2*H*-pyran-3-yl ((2*S*,3*R*)-3-Hydroxy-4-((*N*-isobutyl-1-methyl-1*H*-pyrazole)-4-sulfonamido)-1-phenylbutan-2-yl)­carbamate (**14h**)

The procedure for **11a** with **10b** (10 mg, 0.03 mmol, 1 equiv) and 1-methyl-1*H*-pyrazole-4-sulfonyl chloride (6 mg, 0.03 mmol, 1.1 equiv) to give the title compound as a clear glass after purification by RP-HPLC (20–80% MeCN in 0.05% aqueous NH_4_OH solution over 10 min) (6.3 mg, 45%) was followed. ^1^H NMR (400 MHz, DMSO-*d*_6_) δ 8.32 – 8.28 (m, 1H), 7.79 – 7.75 (m, 1H), 7.29 – 7.18 (m, 4H), 7.17 – 7.10 (m, 2H), 5.05 (d, *J* = 6.3 Hz, 1H), 4.37 – 4.20 (m, 1H), 3.88 (s, 3H), 3.72 – 3.47 (m, 5H), 3.40 – 3.22 (m, 2H), 3.04 – 2.88 (m, 2H), 2.81 – 2.63 (m, 2H), 2.03 – 1.91 (m, 1H), 1.71 – 1.60 (m, 2H), 1.44 – 1.30 (m, 2H), 0.87 (d, *J* = 6.5 Hz, 3H), 0.83 (d, *J* = 6.6 Hz, 3H). ^13^C NMR (101 MHz, DMSO-*d*_6_) δ 155.4, 139.6, 138.1, 132.7, 129.3, 127.9, 125.8, 119.7, 72.0, 69.2, 67.3, 66.9, 57.1, 55.8, 53.0, 40.4, 35.2, 27.8, 26.5, 22.6, 20.1, 20.1. HRMS (TOF, ES+), C_24_H_37_N_4_O_6_S [M + H]^+^ calc. mass 509.2428, found 509.2430.

#### (*R*)-Tetrahydro-2*H*-pyran-3-yl­((2*S*,3*R*)-3-hydroxy-4-((*N*-isobutyl-2,4-dimethylthiazole)-5-sulfonamido)-1-phenylbutan-2-yl)­carbamate (**14i**)

The procedure for **11a** with **10b** (15 mg, 0.04 mmol, 1 equiv) and 2,4-dimethylthiazole-5-sulfonyl chloride (9.6 mg, 0.05 mmol, 1.1 equiv) to give the title compound as a clear glass after purification by column chromatography (0–70% EtOAc in hexanes) (11 mg, 50%) was followed. ^1^H NMR (400 MHz, DMSO-*d*_6_) δ 7.27 – 7.05 (m, 6H), 5.11 (d, *J* = 6.8 Hz, 1H), 4.37 – 4.21 (m, 1H), 3.67 – 3.55 (m, 2H), 3.54 – 3.42 (m, 3H), 3.41 – 3.27 (m, 2H), 3.14 – 3.04 (m, 1H), 3.00 (dd, *J* = 13.8, 3.2 Hz, 1H), 2.95 – 2.84 (m, 2H), 2.64 (s, 3H), 2.52 (s, 3H), 2.08 – 1.95 (m, 1H), 1.71 – 1.59 (m, 2H), 1.44 – 1.13 (m, 2H), 0.86 (d, *J* = 6.5 Hz, 3H), 0.82 (d, *J* = 6.7 Hz, 3H). ^13^C NMR (101 MHz, DMSO-*d*_6_) δ 168.3, 155.3, 154.7, 139.4, 129.1, 129.1, 128.4, 127.9, 127.9, 125.7, 71.9, 69.2, 67.2, 66.8, 56.6, 55.9, 52.2, 35.5, 27.8, 26.1, 22.6, 19.9, 19.8, 18.9, 16.3. HRMS (TOF, ES+), C_25_H_38_N_3_O_6_S_2_ [M + H]^+^ calc. mass 540.2197, found 540.2196.

### Synthesis of Compounds **11b**–**j** (Scheme 2)

#### *tert*-Butyl ((2*S*,3*R*)-3-Hydroxy-4-(*N*-isobutylphenylsulfonamido)-1-phenylbutan-2-yl)­carbamate (**12**)

To a solution of *tert*-butyl ((2*S*,3*R*)-3-hydroxy-4-(isobutylamino)-1-phenylbutan-2-yl)­carbamate (**6**) (100 mg, 0.3 mmol, 1 equiv) in DCM (1.5 mL) was added a solution of NaHCO_3_ (50 mg, 0.59 mmol, 2 equiv) in water (0.75 mL), and the resulting mixture was cooled to 0 °C. Benzenesulfonyl chloride (0.046 mL, 0.36 mmol, 1.2 equiv) was added dropwise, and the reaction was slowly warmed to r.t. and stirred for 16 h. Upon completion, the reaction mixture was diluted with DCM and the layers were separated. The organic layers were washed sequentially with sat. NaHCO_3_ solution and brine, dried over MgSO_4_, filtered, and concentrated. The crude residue was purified by column chromatography (0–40% EtOAc in hexanes) to afford the title compound as a colorless oil that solidified upon standing (105 mg, 64%). ^1^H NMR (400 MHz, MeOD) δ 7.87 – 7.80 (m, 2H), 7.71 – 7.51 (m, 3H), 7.32 – 7.20 (m, 5H), 3.81 – 3.67 (m, 1H), 3.65 – 3.55 (m, 1H), 3.41 (dd, *J* = 15.0, 2.7 Hz, 1H), 3.17 – 2.82 (m, 4H), 2.54 (dd, *J* = 13.8, 10.8 Hz, 1H), 2.09 – 1.94 (m, 1H), 1.29 (s, 9H), 0.97 – 0.79 (m, 6H). ^13^C NMR (101 MHz, MeOD) δ 158.0, 140.6, 140.2, 133.7, 130.4, 130.2, 129.1, 128.5, 127.0, 79.9, 74.2, 58.6, 56.8, 53.8, 37.3, 28.7, 27.9, 20.4, 20.4. HRMS (TOF, ES+), C_25_H_37_N_2_O_5_S [M + H]^+^ calc. mass 477.2418, found 477.2411.

#### *N*-((2*R*,3*S*)-3-Amino-2-hydroxy-4-phenylbutyl)-*N*-isobutylbenzenesulfonamide Hydrochloride (**13**)

To a solution of **12** (50 mg, 0.15 mmol, 1 equiv) in 1,4-dioxane (0.24 mL) was added 4 M HCl solution in 1,4-dioxane (0.26 mL, 1.05 mmol, 10 equiv), and the mixture was stirred at r.t. for 2 h. Upon completion, the reaction mixture was concentrated to dryness to afford the title compound as a pale-yellow oil that solidified upon standing (41 mg, 95%). ^1^H NMR (400 MHz, MeOD) δ 7.87 – 7.79 (m, 2H), 7.73 – 7.54 (m, 3H), 7.43 – 7.37 (m, 4H), 7.35 – 7.28 (m, 1H), 4.14 (td, *J* = 6.6, 2.4 Hz, 1H), 3.82 – 3.69 (m, 1H), 3.51 (dd, *J* = 14.9, 6.3 Hz, 1H), 3.26 (dd, *J* = 14.3, 5.0 Hz, 1H), 3.06 (dd, *J* = 13.8, 8.6 Hz, 1H), 2.98 – 2.73 (m, 3H), 2.01 – 1.86 (m, 1H), 0.92 (d, *J* = 6.6 Hz, 3H), 0.84 (d, *J* = 6.6 Hz, 3H).^13^C NMR (101 MHz, MeOD) δ 139.8, 137.3, 134.1, 130.5, 130.4, 130.1, 128.5, 70.7, 68.1, 59.5, 56.6, 53.0, 33.2, 28.2, 20.4. HRMS (TOF, ES+), C_21_H_29_N_2_O_3_S [M + H]^+^ calc. mass 377.1893, found 377.1902.

#### (*R*)-Tetrahydrofuran-3-yl ((2*S*,3*R*)-3-Hydroxy-4-(*N*-isobutylphenylsulfonamido)-1-phenylbutan-2-yl)­carbamate (**11b**)

(*R*)-Tetrahydrofuran-3-ol (0.012 mL, 0.15 mmol, 3 equiv) was added to a suspension of *N*,*N′*-disuccinimidyl carbonate (19.5 mg, 0.076 mmol, 1.5 equiv) in MeCN (0.5 mL), followed by the addition of pyridine (0.075 mL). The resulting reaction mixture was stirred at r.t. for 1 h, after which time 13 (21 mg, 0.051 mmol, 1 equiv) was added, followed by triethylamine (8.5 μL, 0.061 mmol, 1.2 equiv). The resulting reaction mixture was stirred at r.t. for 1 h, after which time H_2_O was added, and the aqueous layer was extracted with DCM. Combined organic extracts were filtered through a hydrophobic phase separator and concentrated. The crude residue was purified by column chromatography (0–60% EtOAc in hexanes) to give the title compound as a white powder (12.3 mg, 49%). ^1^H NMR (400 MHz, DMSO) δ 7.78 (d, *J* = 8.5 Hz, 2H), 7.71 – 7.55 (m, 3H), 7.24 – 7.10 (m, 5H), 5.09 (d, *J* = 6.7 Hz, 1H), 4.90 (dd, *J* = 6.0, 4.1 Hz, 1H), 3.69 – 3.46 (m, 6H), 3.37 – 3.31 (m, 1H), 3.09 – 2.95 (m, 2H), 2.87 – 2.73 (m, 2H), 2.50 – 2.42 (m, 1H), 2.04 – 1.87 (m, 2H), 1.61 – 1.50 (m, 1H), 0.84 (d, *J* = 6.5 Hz, 3H), 0.78 (d, *J* = 6.5 Hz, 3H).^13^C NMR (101 MHz, DMSO) δ 155.6, 139.5, 139.1, 132.7, 129.3, 129.2, 127.9, 127.1, 125.8, 74.3, 72.5, 72.2, 66.1, 56.6, 55.8, 52.2, 35.3, 32.3, 26.2, 19.9, 19.9. HRMS (TOF, ES+), C_25_H_35_N_2_O_6_S [M + H]^+^ calc. mass 491.2210, found 491.2209.

#### (*S*)-Tetrahydro-2*H*-pyran-3-yl ((2*S*,3*R*)-3-Hydroxy-4-(*N*-isobutylphenylsulfonamido)-1-phenylbutan-2-yl)­carbamate (**11c**)

The procedure for **11b** with **13** (21 mg, 0.051 mmol, 1 equiv) and (*S*)-tetrahydro-2*H*-pyran-3-ol (15.6 mg, 0.15 mmol, 3 equiv) to give the title compound as a white powder after purification by column chromatography (0–60% EtOAc in hexanes) (9.2 mg, 36%) was followed. ^1^H NMR (400 MHz, DMSO-*d*_6_) δ 7.82 – 7.75 (m, 2H), 7.74 – 7.63 (m, 1H), 7.63 – 7.52 (m, 2H), 7.29 – 7.10 (m, 6H), 5.07 (d, *J* = 6.6 Hz, 1H), 4.31 (m, 1H), 3.65 – 3.38 (m, 5H), 3.35 – 3.31 (m, 1H), 3.22 – 3.11 (m, 1H), 3.08 – 2.96 (m, 2H), 2.91 – 2.73 (m, 2H), 2.04 – 1.89 (m, 1H), 1.86 – 1.76 (m, 1H), 1.72 – 1.63 (m, 1H), 1.57 – 1.33 (m, 2H), 0.83 (d, *J* = 6.6 Hz, 3H), 0.78 (d, *J* = 6.6 Hz, 3H).^13^C NMR (101 MHz, DMSO-*d*_6_) δ 155.4, 139.5, 139.2, 132.7, 129.3, 129.2, 128.0, 127.0, 125.8, 72.0, 69.2, 67.3, 66.8, 56.4, 55.8, 52.1, 35.4, 28.1, 26.1, 22.9, 19.9, 19.9. HRMS (TOF, ES+), C_26_H_37_N_2_O_6_S [M + H]^+^ calc. mass 505.2367, found 505.2365.

#### (*R*)-Tetrahydro-2*H*-pyran-3-yl ((2*S*,3*R*)-3-Hydroxy-4-(*N*-isobutylphenylsulfonamido)-1-phenylbutan-2-yl)­carbamate (**11d**)

The procedure for **11b** with **13** (21 mg, 0.051 mmol, 1 equiv) and (*R*)-tetrahydro-2*H*-pyran-3-ol (15.6 mg, 0.15 mmol, 3 equiv) to give the title compound as a clear oil that solidified upon standing after purification by column chromatography (0–100% EtOAc in hexanes) (12.3 mg, 50%) was followed. ^1^H NMR (400 MHz, MeOD) δ 7.87 – 7.80 (m, 2H), 7.67 – 7.51 (m, 3H), 7.24 (d, *J* = 4.5 Hz, 4H), 7.19 – 7.13 (m, 1H), 4.45 – 4.34 (m, 1H), 3.84 – 3.53 (m, 5H), 3.50 (dd, *J* = 11.8, 5.3 Hz, 1H), 3.44 (dd, *J* = 14.9, 3.1 Hz, 1H), 3.19 – 3.03 (m, 2H), 2.96 (dd, *J* = 14.9, 8.6 Hz, 1H), 2.87 (dd, *J* = 13.6, 6.7 Hz, 1H), 2.60 – 2.51 (m, 1H), 2.09 – 1.94 (m, 1H), 1.84 – 1.65 (m, 2H), 1.52 – 1.38 (m, 2H), 0.92 (d, *J* = 6.6 Hz, 3H), 0.86 (d, *J* = 6.6 Hz, 3H).^13^C NMR (101 MHz, MeOD) δ 157.9, 140.5, 140.2, 133.8, 130.5, 130.2, 129.1, 128.5, 127.1, 74.1, 70.8, 69.5, 68.8, 58.7, 57.3, 53.8, 37.1, 29.0, 27.9, 23.7, 20.4, 20.4. HRMS (TOF, ES+), C_26_H_37_N_2_O_6_S [M + H]^+^ calc. mass 505.2367, found 505.2365.

#### Tetrahydro-2*H*-pyran-4-yl ((2*S*,3*R*)-3-Hydroxy-4-(*N*-isobutylphenylsulfonamido)-1-phenylbutan-2-yl)­carbamate (**11e**)

The procedure for **11b** with **13** (21 mg, 0.051 mmol, 1 equiv) and tetrahydro-2*H*-pyran-4-ol (0.015 mL, 0.15 mmol, 3 equiv) to give the title compound as a clear oil that solidified upon standing after purification by column chromatography (0–70% EtOAc in hexanes) (17 mg, 66%) was followed. ^1^H NMR (400 MHz, MeOD) δ 7.87 – 7.80 (m, 2H), 7.70 – 7.53 (m, 3H), 7.27 – 7.21 (m, 4H), 7.19 – 7.13 (m, 1H), 4.62 – 4.54 (m, 1H), 3.89 – 3.65 (m, 4H), 3.57 – 3.39 (m, 3H), 3.20 – 3.04 (m, 2H), 3.01 – 2.84 (m, 2H), 2.55 (dd, *J* = 13.8, 10.9 Hz, 1H), 2.07 – 1.96 (m, 1H), 1.89 – 1.77 (m, 1H), 1.71 – 1.63 (m, 1H), 1.62 – 1.51 (m, 1H), 1.42 – 1.33 (m, 1H), 0.91 (d, *J* = 6.7 Hz, 3H), 0.86 (d, *J* = 6.7 Hz, 3H).^13^C NMR (101 MHz, MeOD) δ 157.9, 140.5, 140.2, 133.8, 130.5, 130.2, 129.1, 128.5, 127.1, 74.1, 70.1, 66.0, 58.7, 57.2, 53.8, 37.2, 33.0, 32.8, 27.9, 20.4, 20.4. HRMS (TOF, ES+), C_26_H_37_N_2_O_6_S [M + H]^+^ calc. mass 505.2367, found 505.2364

#### Cyclopropyl­((2*S*,3*R*)-3-hydroxy-4-(*N*-isobutylphenylsulfonamido)-1-phenylbutan-2-yl)­carbamate (**11f**)

The procedure for **11b** with **13** (21 mg, 0.051 mmol, 1 equiv) and cyclopropanol (8.9 mg, 0.15 mmol, 3 equiv) to give the title compound as a clear oil that solidified upon standing after purification by column chromatography (0–60% EtOAc in hexanes) (9.2 mg, 39%). ^1^H NMR (400 MHz, MeOD) δ 7.87 – 7.79 (m, 2H), 7.64 – 7.53 (m, 3H), 7.31 – 7.06 (m, 5H), 3.83 – 3.75 (m, 2H), 3.72 – 3.64 (m, 1H), 3.43 (dd, *J* = 15.0, 3.0 Hz, 1H), 3.18 – 3.05 (m, 2H), 2.99 – 2.80 (m, 2H), 2.60 – 2.42 (m, 1H), 2.09 – 1.94 (m, 1H), 0.92 (d, *J* = 6.6 Hz, 3H), 0.86 (d, *J* = 6.7 Hz, 3H), 0.64 – 0.35 (m, 4H).^13^C NMR (101 MHz, MeOD) δ 159.2, 140.5, 140.1, 133.8, 130.4, 130.2, 129.2, 128.5, 127.2, 74.1, 58.7, 57.3, 53.8, 40.3, 37.0, 27.9, 20.4, 20.4, 5.6, 5.5. HRMS (TOF, ES+), C_24_H_33_N_2_O_5_S [M + H]^+^ calc. mass 461.2105, found 461.2106.

#### Cyclobutyl­((2*S*,3*R*)-3-hydroxy-4-(*N*-isobutylphenylsulfonamido)-1-phenylbutan-2-yl)­carbamate (**11g**)

The procedure for **11b** with **13** (15 mg, 0.036 mmol, 1 equiv) and cyclobutanol (8.5 μL, 0.11 mmol, 3 equiv) to give the title compound as a clear oil that solidified upon standing after purification by column chromatography (0–50% EtOAc in hexanes) (10.9 mg, 63%) was followed. ^1^H NMR (400 MHz, MeOD) δ 7.87 – 7.79 (m, 2H), 7.68 – 7.52 (m, 3H), 7.29 – 7.12 (m, 5H), 4.68 (p, *J* = 7.4 Hz, 1H), 3.84 – 3.73 (m, 1H), 3.70 – 3.60 (m, 1H), 3.41 (dd, *J* = 15.0, 3.0 Hz, 1H), 3.20 – 3.04 (m, 2H), 3.00 – 2.82 (m, 2H), 2.61 – 2.45 (m, 1H), 2.33 – 2.09 (m, 2H), 2.04 – 1.96 (m, 2H), 1.89 – 1.80 (m, 1H), 1.77 – 1.47 (m, 2H), 0.91 (d, *J* = 6.6 Hz, 3H), 0.86 (d, *J* = 6.7 Hz, 3H). ^13^C NMR (101 MHz, MeOD) δ 158.0, 140.5, 140.2, 133.8, 130.4, 130.2, 129.2, 128.5, 127.1, 74.1, 70.0, 58.7, 57.2, 53.8, 40.3, 37.1, 31.3, 31.2, 27.9, 20.4, 13.9. HRMS (TOF, ES+), C_25_H_35_N_2_O_5_S [M + H]^+^ calc. mass 475.2261, found 475.2263.

#### *N*-((2*S*,3*R*)-3-Hydroxy-4-(*N*-isobutylphenylsulfonamido)-1-phenylbutan-2-yl)­bicyclo­[1.1.1]­pentane-1-carboxamide (**11h**)

To a solution of bicyclo[1.1.1]­pentane-1-carboxylic acid (20 mg, 0.18 mmol, 1 equiv) and DIPEA (0.12 mL, 0.71 mmol, 4 equiv) in DCM (1 mL) was added HATU (81 mg, 0.21 mmol, 1.2 equiv). The resulting solution was stirred at r.t. for 5 min, after which time **13** (81 mg, 0.20 mmol, 1.1 equiv) was added. The resulting reaction mixture was stirred at r.t. for 1 h, after which time H_2_O was added. The aqueous layer was extracted with DCM, and combined organic extracts were filtered through a hydrophobic phase separator and concentrated. Crude residue was purified by column chromatography (0–50% EtOAc in hexanes) to give the title compound as a clear oil that solidified upon standing (69 mg, 82%). ^1^H NMR (400 MHz, MeOD) δ 7.84 – 7.77 (m, 2H), 7.62 – 7.53 (m, 3H), 7.28 – 7.11 (m, 5H), 3.98 – 3.77 (m, 2H), 3.37 (dd, *J* = 15.1, 2.8 Hz, 1H), 3.20 (dd, *J* = 13.7, 3.6 Hz, 1H), 3.10 (dd, *J* = 13.6, 8.6 Hz, 1H), 2.93 – 2.78 (m, 2H), 2.64 (dd, *J* = 13.8, 11.2 Hz, 1H), 2.09 – 1.94 (m, 1H), 1.91 (s, 6H), 0.92 (d, *J* = 6.5 Hz, 3H), 0.85 (d, *J* = 6.7 Hz, 3H).^13^C NMR (101 MHz, MeOD) δ 172.4, 140.5, 140.1, 133.8, 130.4, 130.2, 129.1, 128.4, 127.2, 74.4, 58.9, 55.4, 54.2, 51.9, 45.3, 40.3, 36.6, 27.9, 27.6, 20.4. HRMS (TOF, ES+), C_26_H_35_N_2_O_4_S [M + H]^+^ calc. mass 471.2312, found 471.2309.

#### *N*-((2*S*,3*R*)-3-Hydroxy-4-(*N*-isobutylphenylsulfonamido)-1-phenylbutan-2-yl)-1-methylcyclopropane-1-carboxamide (**11i**)

The procedure for **11h** with **13** (15 mg, 0.04 mmol, 1 equiv) and 1-methylcyclopropane-1-carboxylic acid (4.8 mg, 0.048 mmol, 1.2 equiv) to give the title compound as a clear oil that solidified upon standing after purification by RP-HPLC (30–60% MeCN in 0.1% aqueous TFA solution over 5 min) (7.5 mg, 41%) was followed. ^1^H NMR (400 MHz, MeOD) δ 7.85 – 7.77 (m, 2H), 7.69 – 7.53 (m, 3H), 7.30 – 7.12 (m, 5H), 4.05 – 3.94 (m, 1H), 3.84 (td, *J* = 8.3, 2.8 Hz, 1H),D 3.36 (dd, *J* = 15.1, 2.8 Hz, 1H), 3.19 (dd, *J* = 13.8, 3.8 Hz, 1H), 3.11 (dd, *J* = 13.5, 8.6 Hz, 1H), 2.93 – 2.79 (m, 2H), 2.70 (dd, *J* = 13.8, 11.1 Hz, 1H), 2.10 – 1.95 (m, 1H), 1.22 (s, 3H), 0.92 (d, *J* = 6.5 Hz, 3H), 0.89 – 0.77 (m, 5H), 0.56 – 0.40 (m, 2H).^13^C NMR (101 MHz, MeOD) δ 177.4, 140.4, 140.2, 133.8, 130.4, 130.2, 129.2, 128.4, 127.1, 74.5, 58.9, 55.7, 54.2, 40.3, 36.6, 27.9, 20.4, 20.4, 19.9, 16.1, 15.8. HRMS (TOF, ES+), C_25_H_35_N_2_O_4_S [M + H]^+^ calc. mass 459.2312, found 459.2314.

#### *N*-((2*S*,3*R*)-3-Hydroxy-4-(*N*-isobutylphenylsulfonamido)-1-phenylbutan-2-yl)-1-methylcyclobutane-1-carboxamide (**11j**)

The procedure for **11h** with 13 (15 mg, 0.04 mmol, 1 equiv) and 1-methylcyclobutane-1-carboxylic acid (5.5 mg, 0.048 mmol, 1.2 equiv) to give the title compound as a clear oil that solidified upon standing after purification by RP-HPLC (32–62% MeCN in 0.1% aqueous TFA solution over 5 min) (7.9 mg, 42%) was followed. ^1^H NMR (400 MHz, MeOD) δ 7.86 – 7.77 (m, 2H), 7.70 – 7.51 (m, 3H), 7.25 – 7.20 (m, 4H), 7.20 – 7.12 (m, 1H), 4.07 – 3.95 (m, 1H), 3.80 (td, *J* = 8.7, 2.7 Hz, 1H), 3.40 (dd, *J* = 15.0, 2.7 Hz, 1H), 3.25 (dd, *J* = 13.9, 3.7 Hz, 1H), 3.10 (dd, *J* = 13.6, 8.4 Hz, 1H), 3.01 – 2.83 (m, 2H), 2.65 – 2.59 (m, 1H), 2.22 – 1.97 (m, 3H), 1.94 – 1.76 (m, 1H), 1.73 – 1.62 (m, 2H), 1.60 – 1.49 (m, 1H), 1.13 (s, 3H), 0.91 (d, *J* = 6.6 Hz, 3H), 0.86 (d, *J* = 6.7 Hz, 3H).^13^C NMR (101 MHz, MeOD) δ 181.3, 140.7, 140.2, 133.8, 130.4, 130.2, 129.1, 128.4, 127.1, 74.5, 58.7, 55.0, 54.1, 45.4, 40.3, 36.9, 32.2, 32.2, 27.9, 25.4, 20.4, 15.2. HRMS (TOF, ES+), C_26_H_37_N_2_O_4_S [M + H]^+^ calc. mass 473.2469, found 473.2466.

### Synthesis of Compounds **21a**–**e** (Scheme 3)

#### *tert*-Butyl (*S*)-(4-Hydroxy-1-phenylbutan-2-yl)­carbamate (**16**)

A solution of (*S*)-3-((*tert*-butoxycarbonyl)­amino)-4-phenylbutanoic acid (**15**) (790 mg, 2.83 mmol, 1 equiv) in THF (15 mL) was cooled to 0 °C, and LAH (5.66 mL, 5.66 mmol, 2 equiv, 1 M solution in diethyl ether) was added dropwise under an N_2_ atmosphere. The resulting solution was warmed to r.t. and stirred for 1 h, after which time the reaction mixture was cooled to 0 °C and diluted with diethyl ether. Sequentially, 0.20 mL of H_2_O, 0.20 mL of 4 M NaOH solution, and 0.60 mL of H_2_O were added, and the reaction mixture was warmed to r.t. and stirred for 15 min. MgSO_4_ was then added, and the resulting reaction mixture was stirred for an additional 15 min. Solids were removed by filtration with DCM, and the filtrate was concentrated to give the title compound as a slightly yellow oil, which was dried and used without additional purification (656 mg, 87%). ^1^H NMR (400 MHz, CDCl_3_) δ 7.33 – 7.28 (m, 2H), 7.25 – 7.17 (m, 3H), 4.52 – 4.42 (m, 1H), 4.15 – 4.05 (m, 1H), 3.66 – 3.58 (m, 2H), 2.81 (d, *J* = 6.7 Hz, 2H), 1.89 – 1.81 (m, 1H), 1.41 (s, 9H), 1.37 – 1.27 (m, 1H). ^13^C NMR (101 MHz, CDCl_3_) δ 157.1, 137.7, 129.4, 128.7, 126.7, 80.1, 58.9, 47.9, 41.6, 38.0, 28.4. HRMS (TOF, ES+), C_15_H_23_NO_3_Na [M + Na]^+^ calc. mass 288.1570, found 288.1571.

#### *tert*-Butyl (*S*)-(4-oxo-1-Phenylbutan-2-yl)­carbamate (**17**)

To a solution of **16** (656 mg, 2.47 mmol, 1 equiv) in DCM (10 mL) and DMSO (5 mL) was added DIPEA (1.29 mL, 7.41 mmol, 3 equiv). The resulting solution was cooled to 0 °C, and sulfur trioxide pyridine complex (590 mg, 3.71 mmol, 1.5 equiv) was added. The resulting reaction mixture was warmed to r.t. and stirred for 1.5 h, after which time the reaction mixture was diluted with H_2_O and EtOAc. The organic layer was washed 3× with brine and dried over MgSO_4_. Solvents were filtered and concentrated, and the crude title compound was used directly without further purification (651 mg, 100%).

#### *tert*-Butyl (*S*)-(4-(Isobutylamino)-1-phenylbutan-2-yl)­carbamate (**18**)

To a solution of **17** (965 mg, 3.66 mmol, 1 equiv) and isobutylamine (1.09 mL, 11.0 mmol, 3 equiv) in DCM (20 mL) was added sodium triacetoxyborohydride (2.33 g, 11.0 mmol, 3 equiv) in one portion. The resulting reaction mixture was stirred at r.t. overnight, after which time the reaction was quenched with the addition of sat. NaHCO_3_ solution and extracted with DCM. Combined organic extracts were dried over MgSO_4_, and solvents were filtered and concentrated to give the title compound as a yellow oil (1.03 g, 88%). ^1^H NMR (400 MHz, CDCl_3_) δ 7.30 – 7.26 (m, 2H), 7.23 – 7.16 (m, 3H), 5.38 (d, *J* = 8.2 Hz, 1H), 3.95 – 3.83 (m, 1H), 2.90 (dd, *J* = 13.5, 5.6 Hz, 1H), 2.75 – 2.62 (m, 3H), 2.44 – 2.33 (m, 2H), 1.79 – 1.69 (m, 2H), 1.40 (s, 9H), 1.44 – 1.36 (m, 1H), 0.90 (d, *J* = 6.6 Hz, 6H). ^13^C NMR (101 MHz, CDCl_3_) δ 156.0, 138.3, 129.6, 128.5, 126.4, 79.1, 58.0, 50.6, 46.7, 41.5, 33.2, 28.5, 28.2, 20.8, 20.8. HRMS (TOF, ES+), C_19_H_33_N_2_O_2_ [M + H]^+^ calc. mass 321.2537, found 321.2543.

#### *tert*-Butyl (*S*)-(4-(*N*-Isobutylphenylsulfonamido)-1-phenylbutan-2-yl)­carbamate (**19**)

To a solution of **18** (1.03 g, 3.21 mmol, 1 equiv) in DCM (8 mL) was added a solution of NaHCO_3_ (539 mg, 6.42 mmol, 2 equiv) in H_2_O (6 mL). A solution of benzenesulfonyl chloride (0.49 mL, 3.85 mmol, 1.2 equiv) in DCM (4 mL) was then added dropwise. The resulting reaction mixture was stirred at r.t. overnight, after which time the reaction was diluted with DCM and H_2_O, and the aqueous layer was extracted with DCM. Combined organic extracts were filtered through a hydrophobic phase separator and concentrated. The crude residue was purified by column chromatography (0–8% MeOH in DCM) to give the title compound as a yellow oil (1.29 g, 87%). ^1^H NMR (400 MHz, CDCl_3_) δ 7.78 – 7.73 (m, 2H), 7.57 – 7.53 (m, 1H), 7.49 – 7.45 (m, 2H), 7.31 – 7.20 (m, 3H), 7.13 (d, *J* = 7.4 Hz, 2H), 4.42 (d, *J* = 9.1 Hz, 1H), 3.75 – 3.62 (m, 1H), 3.22 – 3.10 (m, 1H), 3.04 – 3.00 (m, 1H), 2.90 – 2.68 (m, 4H), 1.84 – 1.68 (m, 2H), 1.59 – 1.53 (m, 1H), 1.39 (s, 9H), 0.87 – 0.84 (m, 6H). ^13^C NMR (101 MHz, CDCl_3_) δ 155.6, 139.5, 137.7, 132.5, 129.5, 129.1, 128.6, 127.2, 126.6, 79.4, 56.9, 50.0, 46.6, 41.6, 34.1, 28.5, 27.3, 20.1, 20.1. HRMS (TOF, ES+), C_25_H_36_N_2_O_4_SNa [M + Na]^+^ calc. mass 483.2288, found 483.2286.

#### (*S*)-*N*-(3-Amino-4-phenylbutyl)-*N*-isobutylbenzenesulfonamide Hydrochloride (**20**)

To a stirring solution of **19** (1.28 g, 2.78 mmol, 1 equiv) in 1,4-dioxane (15 mL) was added 4 M HCl in 1,4-dioxane solution (15 mL) dropwise. The resulting reaction mixture was stirred at r.t. for 2 h, after which time solvents were concentrated, and the resulting white solid was dried and used without further purification (1.10 g, 100%). ^1^H NMR (400 MHz, MeOD) δ 7.84 – 7.80 (m, 2H), 7.68 – 7.64 (m, 1H), 7.60 – 7.56 (m, 2H), 7.40 – 7.36 (m, 2H), 7.34 – 7.28 (m, 3H), 3.64 – 3.57 (m, 1H), 3.35 – 3.27 (m, 1H), 3.17 – 3.10 (m, 1H), 3.01 (dd, *J* = 13.6, 6.1 Hz, 1H), 2.93 (dd, *J* = 13.6, 8.3 Hz, 1H), 2.81 (dd, *J* = 13.6, 8.2 Hz, 1H), 2.73 (dd, *J* = 13.5, 7.0 Hz, 1H), 1.97 – 1.79 (m, 2H), 1.69 – 1.59 (m, 1H), 0.77 (d, *J* = 6.6 Hz, 3H), 0.73 (d, *J* = 6.6 Hz, 3H). ^13^C NMR (101 MHz, MeOD) δ 140.2, 137.0, 134.1, 130.6, 130.4, 130.1, 128.6, 128.3, 58.6, 51.9, 46.8, 40.0, 33.2, 28.3, 20.3, 20.2. HRMS (TOF, ES+), C_20_H_29_N_2_O_2_S [M + H]^+^ calc. mass 361.1944, found 361.1947.

#### (*S*)-Tetrahydro-2*H*-pyran-3-yl ((*S*)-4-(*N*-Isobutylphenylsulfonamido)-1-phenylbutan-2-yl)­carbamate (**21a**)

(*S*)-Tetrahydro-2*H*-pyran-3-ol (11.6 mg, 0.11 mmol, 3 equiv) was added to a suspension of *N*,*N′*-disuccinimidyl carbonate (12.5 mg, 0.057 mmol, 1.5 equiv) in MeCN (0.5 mL), followed by the addition of pyridine (0.075 mL). The resulting reaction mixture was stirred at r.t. for 1 h, after which time **20** (15 mg, 0.038 mmol, 1 equiv) and triethylamine (6.3 μL, 0.045 mmol, 1.2 equiv) were added. The resulting reaction mixture was stirred at r.t. for 1 h, after which time the reaction mixture was quenched with H_2_O and extracted with DCM. Combined organic extracts were filtered through a hydrophobic phase separator and concentrated. The crude residue was purified by RP-HPLC (30–70% MeCN in 0.1% aqueous TFA solution over 5 min). Fractions containing the product were basified with sat. NaHCO_3_ solution and extracted with DCM. Combined organic extracts were filtered through a hydrophobic phase separator and concentrated to give the title compound as a colorless oil (7.0 mg, 38%). ^1^H NMR (400 MHz, MeOD) δ 7.79 – 7.75 (m, 2H), 7.65 – 7.60 (m, 1H), 7.58 – 7.53 (m, 2H), 7.29 – 7.23 (m, 2H), 7.21 – 7.15 (m, 3H), 4.52 – 4.45 (m, 1H), 3.73 – 3.54 (m, 4H), 3.41 (dd, *J* = 11.6, 5.9 Hz, 1H), 3.16 – 3.10 (m, 2H), 2.84 (d, *J* = 7.5 Hz, 2H), 2.75 – 2.64 (m, 2H), 1.96 – 1.65 (m, 5H), 1.63 – 1.48 (m, 2H), 0.87 (d, *J* = 4.5 Hz, 3H), 0.85 (d, *J* = 4.5 Hz, 3H). ^13^C NMR (101 MHz, MeOD) δ 157.9, 140.9, 139.8, 133.8, 130.4, 130.3, 129.4, 128.2, 127.4, 70.9, 69.5, 68.8, 57.6, 52.1, 47.3, 42.4, 34.5, 29.4, 28.2, 24.1, 20.4, 20.4. HRMS (TOF, ES+), C_26_H_37_N_2_O_5_S [M + H]^+^ calc. mass 489.2418, found 489.2415.

#### Cyclopropyl (*S*)-(4-(*N*-Isobutylphenylsulfonamido)-1-phenylbutan-2-yl)­carbamate (**21b**)

The procedure for **21a** with cyclopropanol (6.6 mg, 0.11 mmol, 3 equiv) and **20** (15 mg, 0.038 mmol, 1 equiv) to give the title compound as a white solid after purification by RP-HPLC (30–70% MeCN in 0.1% aqueous TFA solution over 5 min) (8.2 mg, 49%) was followed. ^1^H NMR (400 MHz, MeOD) δ 7.79 – 7.74 (m, 2H), 7.65 – 7.61 (m, 1H), 7.59 – 7.53 (m, 2H), 7.29 – 7.24 (m, 2H), 7.22 – 7.18 (m, 1H), 7.18 – 7.13 (m, 2H), 3.88 – 3.84 (m, 1H), 3.73 – 3.63 (m, 1H), 3.12 (t, *J* = 7.9 Hz, 2H), 2.89 – 2.79 (m, 2H), 2.74 – 2.64 (m, 2H), 1.82 – 1.69 (m, 2H), 1.62 – 1.52 (m, 1H), 0.87 (d, *J* = 2.6 Hz, 3H), 0.85 (d, *J* = 2.6 Hz, 3H), 0.65 – 0.48 (m, 4H). ^13^C NMR (101 MHz, MeOD) δ 159.2, 140.8, 139.7, 133.8, 130.4, 130.3, 129.4, 128.2, 127.4, 57.6, 52.1, 49.8, 47.3, 42.3, 34.6, 28.2, 20.4, 20.3, 5.6, 5.6. HRMS (TOF, ES+), C_24_H_33_N_2_O_4_S [M + H]^+^ calc. mass 445.2156, found 445.2160.

#### (*S*)-*N*-(4-(*N*-Isobutylphenylsulfonamido)-1-phenylbutan-2-yl)-1-methylcyclopropane-1-carboxamide (**21c**)

To a solution of 1-methylcyclopropane-1-carboxylic acid (3.5 mg, 0.035 mmol, 1 equiv) and DIPEA (0.024 mL, 0.14 mmol, 4 equiv) in DMF (1 mL) was added HATU (16.0 mg, 0.042 mmol, 1.2 equiv). The resulting reaction mixture was stirred at r.t. for 5 min, after which time **20** (15.3 mg, 0.039 mmol, 1.1 equiv) was added. The resulting reaction mixture was stirred at r.t. for 1 h, after which time the reaction mixture was purified directly by RP-HPLC (35–85% MeCN in 0.1% aqueous TFA solution over 5 min). Fractions containing the product were basified with sat. NaHCO_3_ solution and extracted with EtOAc. Combined organic extracts were filtered through a hydrophobic phase separator and concentrated to give the title compound as a white solid (9.7 mg, 63%). ^1^H NMR (400 MHz, MeOD) δ 7.78 – 7.74 (m, 2H), 7.65 – 7.61 (m, 1H), 7.58 – 7.53 (m, 2H), 7.28 – 7.23 (m, 2H), 7.21 – 7.15 (m, 3H), 4.07 – 3.98 (m, 1H), 3.12 – 3.00 (m, 2H), 2.92 – 2.79 (m, 2H), 2.78 – 2.72 (m, 2H), 1.84 – 1.66 (m, 3H), 1.26 (s, 3H), 0.98 – 0.90 (m, 2H), 0.88 (s, 3H), 0.86 (s, 3H), 0.58 – 0.49 (m, 2H). ^13^C NMR (101 MHz, MeOD) δ 177.5, 140.7, 139.8, 133.8, 130.4, 130.3, 129.3, 128.2, 127.4, 57.8, 50.7, 47.6, 41.8, 34.9, 28.3, 20.4, 20.4, 20.3, 20.0, 16.1, 15.9. HRMS (TOF, ES+), C_25_H_35_N_2_O_3_S [M + H]^+^ calc. mass 443.2363, found 443.2365.

#### (*S*)-*N*-(4-(*N*-Isobutylphenylsulfonamido)-1-phenylbutan-2-yl)-1-methylcyclobutane-1-carboxamide (**21d**)

The procedure for **21c** with **20** (20 mg, 0.050 mmol, 1 equiv) and 1-methylcyclobutane-1-carboxylic acid (6.0 mg, 0.061 mmol, 1.2 equiv) to give the title compound as colorless oil after purification by RP-HPLC (37–67% MeCN in 0.1% aqueous TFA solution over 5 min) (10.7 mg, 47%) was followed. ^1^H NMR (400 MHz, MeOD) δ 7.79 – 7.75 (m, 2H), 7.65 – 7.61 (m, 1H), 7.58 – 7.53 (m, 2H), 7.27 – 7.23 (m, 2H), 7.20 – 7.15 (m, 3H), 4.07 – 4.00 (m, 1H), 3.15 – 3.02 (m, 2H), 2.93 – 2.66 (m, 4H), 2.26 – 2.17 (m, 2H), 1.95 – 1.57 (m, 7H), 1.24 (s, 3H), 0.89 (d, *J* = 1.3 Hz, 3H), 0.87 (d, *J* = 1.2 Hz, 3H). ^13^C NMR (101 MHz, MeOD) δ 181.3, 140.8, 139.8, 133.8, 130.4, 130.3, 129.3, 128.2, 127.4, 57.9, 49.9, 47.8, 45.5, 41.9, 35.4, 32.4, 32.3, 28.4, 25.6, 20.4, 20.3, 15.3. HRMS (TOF, ES+), C_26_H_37_N_2_O_3_S [M + H]^+^ calc. mass 457.2519, found 457.2522.

#### (*S*)-*N*-(4-(*N*-Isobutylphenylsulfonamido)-1-phenylbutan-2-yl)­pivalamide (**21e**)

The procedure for **21c** with **20** (15.3 mg, 0.039 mmol, 1.1 equiv) and pivalic acid (3.6 mg, 0.035 mmol, 1 equiv) to give the title compound as a white solid after purification by RP-HPLC (35–85% MeCN in 0.1% aqueous TFA solution over 5 min) (9.4 mg, 60%) was followed. ^1^H NMR (400 MHz, CDCl_3_) δ 7.75 – 7.71 (m, 2H), 7.57 – 7.53 (m, 1H), 7.50 – 7.45 (m, 2H), 7.31 – 7.26 (m, 2H), 7.24 – 7.19 (m, 1H), 7.16 – 7.12 (m, 2H), 5.57 (d, *J* = 8.6 Hz, 1H), 4.11 – 4.02 (m, 1H), 3.13 – 3.06 (m, 1H), 3.03 – 2.95 (m, 1H), 2.90 – 2.78 (m, 4H), 1.95 – 1.86 (m, 1H), 1.79 – 1.68 (m, 2H), 1.10 (s, 9H), 0.87 (d, *J* = 6.6 Hz, 3H), 0.84 (d, *J* = 6.6 Hz, 3H). %). ^13^C NMR (101 MHz, CDCl_3_) δ 178.4, 139.3, 137.7, 132.6, 129.5, 129.2, 128.6, 127.1, 126.7, 57.3, 48.3, 46.8, 41.1, 38.8, 34.7, 27.6, 27.4, 20.1, 20.0. HRMS (TOF, ES+), C_25_H_37_N_2_O_3_S [M + H]^+^ calc. mass 445.2519, found 445.2521.

### Molecular Pharmacology Experimental Conditions

#### Cell Culture

The cell lines used in this manuscript were generated in-house. Their preparation is described in detail in Qi et al., 2024.^34^ Cells were used for 10 passages for experiments, and day-to-day signals in both the CB_2_ and CB_1_ lines were stable across this passage number. Human Embryonic Kidney (HEK) 293 cells stably expressing rCB_2_ or rCB_1_ and the G protein inwardly rectifying potassium channel (GIRK) were maintained in DMEM/F12 containing 10% FBS, 1× antibiotic/antimycotic, 20 mM HEPES, 1 mM sodium pyruvate, 2 mM l-glutamine, 1× nonessential amino acids, 700 μg/mL G418 sulfate, and 0.6 μg/mL puromycin. Cells were monitored by periodic PCR detection using the LookOut Mycoplasma PCR Detection Kit (Sigma-Aldrich, St Louis, Missouri) to eliminate potential mycoplasma infection. Reagents were obtained from Invitrogen (Waltham, Massachusetts) unless otherwise noted.

#### Thallium Flux Assay

Thallium flux assays were performed as previously described in Niswender et al., 2008.^53^ HEK/GIRK cells stably expressing rCB_1_ or rCB_2_ (15,000 cells/20 μL/well) were seeded in 384-well, poly-d-lysine-coated assay plates (Corning Biocoat) (Corning Inc., Corning, New York) and incubated overnight at 37 °C in the presence of 5% CO_2_. The next day, medium was removed and replaced with 20 μL of 1.2 μM thallium-sensitive dye Thallos-AM (ION Biosciences, San Marcos, Texas), prepared as a DMSO stock solution mixed in a 1:1 ratio with 10% (w/v) pluronic acid F-127 and diluted in assay buffer (Hank’s balanced salt solution, 20 mM HEPES, pH 7.4). Following 1 h at room temperature, dye solution was removed and replaced with 20 μL of assay buffer. For concentration–response curve experiments, compounds were serially diluted 1:3 into 10-point concentration response curves in DMSO, transferred to daughter plates using the Echo acoustic plate reformatter (Labcyte, Sunnyvale, California) or Bravo Automated Liquid Handling Platform (Agilent Technologies, Santa Clara, California), and diluted in assay buffer to a 2× final concentration. Thallium flux was measured using the Hamamatsu FDSS/μCELL Kinetic Plate Imager (Bridgewater, New Jersey). After establishment of a fluorescence baseline (excitation, 480 ± 20 nm; emission, 540 ± 30 nm), 20 μL (2×) of the test compound was added to the cells at 1 s and the response was measured. 140 s later, 10 μL (5×) of an EC_20_ concentration of agonist (2-AG) in thallium stimulus buffer (125 mM NaHCO_3_, 1.8 mM CaSO_4_, 1 mM MgSO_4_, 5 mM glucose, 12 mM Tl_2_SO_4_, 10 mM HEPES, 0.5% BSA, pH 7.4) was added to the cells, and the response of the cells was measured for an additional 159 s (data were acquired for 300 s total at 0.5 Hz for 140 s and 1 Hz for 160 s). Raw kinetic data were analyzed in a multistep process. (1) Fluorescence readings for each time point in a well were divided by the fluorescence reading at the initial time point to account for differences in cell number, nonuniform illumination, and dye loading. (2) The slope value for each kinetic trace was calculated for the time window of 145–155 s, a window occurring directly after the second addition. (3) The average slope was calculated for wells containing vehicle, and this value was subtracted from all wells. (4) The vehicle-subtracted slope was normalized to the relevant maximal agonist signal for each assay. For concentration response curves, normalized data were fit to a four-parameter logistic equation using GraphPad Prism (La Jolla, California) or the Dotmatics software platform (Dotmatics, Bishop’s Stortford, UK).

$$y=bottom+\frac{top-bottom}{1+10^{\left(\log\;EC50-A\right)Hillslope}}$$

where *A* is the molar concentration of the compound; *bottom* and *top* denote the lower and upper plateaus of the concentration–response curve, respectively; Hillslope is the Hill coefficient that describes the steepness of the curve; and EC_50_ is the molar concentration of compound required to generate a response halfway between the *top* and *bottom*. Data shown represent the mean ± standard error of the pEC_50_ or maximal response. Experiments were performed in duplicate or triplicate and repeated a minimum of three separate times.

#### PI Hydrolysis Assay

One day prior to experimentation, selected HEK/G_qi9_ monoclonal cells stably expressing rat CB_2_ were plated onto poly-d-lysine-coated clear-bottom 384-well plates (15,000 cells per well) in DMEM supplemented with 10% FBS and 20 mM HEPES. Ten minutes prior to the assay, cell culture medium was replaced with 20 μL of 37 °C Hank’s balanced salt solution (HBSS, with Ca^2+^, Mg^2+^, and glucose). IP_1_ stimulation was then initiated by adding 5 μL of HBSS plus 40 mM Li^+^, and cells were incubated for an additional hour before aspiration and addition of lysis buffer. IP_1_ levels were determined using the Cisbio HTRF IP-ONE assay kit per the manufacturer’s instructions, and fluorescence was measured using an Envision plate reader (PerkinElmer, Waltham, Massachusetts). Data were acquired as HTRF ratio (665/620) and expressed as nanomolar levels of IP_1_.

#### CB_2_ Human Cannabinoid GPCR Cell-Based Agonist Arrestin LeadHunter Assay

This assay was performed at Eurofins (item: 86-001P-2120AG) as detailed: https://www.eurofinsdiscovery.com/catalog/cb2-human-cannabinoid-gpcr-cell-based-agonist-arrestin-leadhunter-assay-us/86-0001P-2120AG

#### CB_2_ Human Cannabinoid GPCR Cell-Based cAMP LeadHunter Assay

This assay was performed at Eurofins (item: 86-001P-2818AG) as detailed: https://www.eurofinsdiscovery.com/catalog/cb2-human-cannabinoid-gpcr-cell-based-agonist-camp-leadhunter-assay-us/86-0007P-2818AG

#### Radioligand Binding Assays

Membranes were made from HEK/GIRK cells stably expressing rat CB_2_. Radioligand competition binding assays were performed as previously described^34^ with minor modifications. In brief, compounds were serially diluted into assay buffer with 0.1% bovine serum albumin (BSA) and added to each well of a 96-well plate, along with 20 μg/well cell membrane and approximately 500 pM [^3^H]-CP55,940 (specific activity = 104 Ci/mmol, PerkinElmer, Waltham, Massachusetts). Following a 3 h incubation period on a shaker at room temperature, the membrane-bound ligand was separated from the free ligand by filtration through glass fiber 96-well filter plates (Unifilter-96, GF/B; PerkinElmer, Waltham, Massachusetts). Forty microliters of scintillation fluid was added to each well, and the membrane-bound radioactivity was determined by scintillation counting (Microbeta2; Revvity, Waltham, Massachusetts). Nonspecific binding was determined using 10 μM of cold CP55,940.

### *In Vitro* DMPK Experimental Conditions

#### Plasma Protein Binding

Determination of fraction unbound (*f*_u_) in plasma from rat and human was conducted *in vitro* via equilibrium dialysis using HTDialysis membrane plates. The top half of the plate was filled with 100 μL of Dulbecco’s phosphate-buffered saline, pH 7.4 (DPBS). The compound was diluted into plasma from each species (5 μM final concentration), which was aliquoted in triplicate to the “bottom half” of the prepared HTD plate wells. The HTD plate was sealed and incubated for 6 h at 37 °C. Following incubation, each well (both top and bottom halves) was transferred (20 μL) to the corresponding wells of a 96-shallow-well (V-bottom) plate. The daughter plates were then matrix-matched (DPBS side wells received equal volume of plasma, and plasma side wells received equal volumes of DPBS), and extraction solution (120 μL; acetonitrile containing 50 nM carbamazepine as internal standard) was added to all wells of both daughter plates to precipitate protein and extract test article. The plates were then sealed and centrifuged (3500 rcf) for 10 min at ambient temperature. Supernatant (60 μL) from each well of the daughter plates was then transferred to the corresponding wells of new daughter plates (96-shallow-well, V bottom) containing water (Milli-Q, 60 μL/well), and the plates were sealed in preparation for LC-MS/MS analysis as follows.

Prepared samples were injected (10 μL each) onto an AB Sciex Triple Quad 4500 mass spectrometer system with an Agilent 1260 Infinity II pump and autosampler. MS parameters were as follows: capillary voltage 5500 V, probe temperature 500 °C, column Fortis C18 (50 × 3.0 mm, 3 μm), column temperature 45 °C, flow rate 0.5 mL/min, default gradient 5% to 95% CH_3_CN (0.5% FA) in water (0.5% FA) over 0.8 min, and hold at 95% CH_3_CN for 0.7 min. Quantitation was performed via AB Sciex MultiQuant software using the raw analyte:internal standard (IS) peak area ratios. The typical detection range for the compounds was 0.5 to ≥5000 ng/mL utilizing a quadratic equation regression with 1/×2 weighting.

The unbound fraction (*f*_u_) was calculated following the equation [mean DPBS well ratio/mean plasma well ratio], and mean values for each species were calculated from three replicates.

#### Predicted Microsomal Clearance

Human and rat hepatic microsomes (0.5 mg/mL) and 1 μM test compound were incubated in 100 mM potassium phosphate pH 7.4 buffer with 3 mM MgCl_2_ at 37 °C with constant shaking. After a 5 min preincubation, the reaction was initiated by addition of NADPH (1 mM). At selected time intervals (0, 3, 7, 15, 25, and 45 min), aliquots were taken and subsequently placed into a 96-well plate containing cold acetonitrile with internal standard (50 nM carbamazepine). Plates were then centrifuged at 3000 RCF (4 °C) for 10 min, and the supernatant was transferred to a separate 96-well plate and diluted 1:1 with water for LC/MS/MS analysis. The *in vitro* half-life (*t*_1/2_, min), intrinsic clearance (CL_int_, mL/min/kg), and subsequent predicted hepatic clearance (CL_hep_, mL/min/kg) were determined using eqs 1–3:

$$T_{1/2}=\frac{\ln\left(2\right)}{k}$$ 1

Equation 1 shows the determination of half-life. *k* represents the slope from linear regression analysis of the natural log percent remaining of test compound as a function of incubation time.

$${CL}_{int}=\frac{0.693}{\mathrm{in}\;vitro\;T_{1/2}}\times \frac{1\;mL\;incubation}{0.5\;mg\;microsomes}\times \frac{45\;mg\;microsomes}{1\;gram\;liver}\times \frac{gram\;liver}{kg\;body\;wt}$$ 2

Equation 2 shows the determination of intrinsic clearance. Scale-up factors (gm liver/kg body weight) of 20 (human) and 45 (rat) were used in this calculation.

$${CL}_{hep}=\frac{{Q_{h}}^{*}{CL}_{int}}{Q_{h}+{CL}_{int}}$$ 3

Equation 3 shows the determination of predicted hepatic clearance. *Q*_h_ represents hepatic blood flow (mL/min/kg): 21 for human, 70 for rat.

### P-gp Efflux

#### Cell Culture

In-house MDCKII-MDR1 cells were cultured in media consisting of Dulbecco’s Modified Eagle’s Media (low glucose), 25 mM HEPES, 10% fetal bovine serum, 1% nonessential amino acids, 100 units/mL penicillin/streptomycin and 4 mM G418 at 37 °C, 5% CO_2_, and 85% relative humidity. On day 1, MDCKII-MDR1 cells were seeded at a density of 45,000 cells/well onto a Corning (Corning, New York) 24-well transwell plate (0.4 μm pore size, 0.33 cm^2^ growth area) and placed in the cell culture incubators. The assay was performed on day 5. The transwell plates received fresh media change 1 day before the experiments to prevent cell starvation.

#### P-Glycoprotein Transwell Assay

All transwell assays were performed in HBSS buffer. Transwell assays were performed at 5 μM concentration of compounds. Transporter studies were initiated by adding dosing solutions into donor compartments and measuring appearance of compounds in receiver compartments after 120 min. Before incubation, donor and receiver samples at 0 min were collected, and after 120 min of incubation, samples were collected for both donor and receiver chambers and crashed with cold acetonitrile containing internal standard (50 nM carbamazepine). The plates were then centrifuged at 3000 RCF (4 °C) for 10 min, and the supernatant was transferred to a separate 96-well plate and diluted 1:1 with water and 0.2% formic acid for LC/MS/MS analysis. The cell monolayer integrity during the incubation with the test compounds were carried out after 120 min assay duration by measuring lucifer yellow (100 μM) fluorescence in both donor and receiver chambers. Quinidine and propranolol were used as P-gp substrate and high passive permeability control, respectively. All the data for controls were withing the acceptable range. The % recovery for the test compounds was above 80%.

#### Data Analysis

To determine apparent permeability (*P*_app_), the following equation was used:

$$P_{app}=\frac{dQ}{dt}\times \frac{1}{\left(A\times C_{0}\right)}$$

where d*Q*/d*t* is the rate of appearance of the test compounds in the receiver compartment, *A* is the surface area of the membrane (0.33 cm^2^), and *C*_0_ is the initial concentration (0 min) of the test compounds in the donor compartment.

The ER was calculated by the following equation:

$$EffluxRatio=\frac{P_{app,B-A}}{P_{app,A-B}}$$

where *P*_app,B‑A_ or *P*_app,A‑B_ refers to the permeability in direction of basolateral to apical (B-to-A) apical to basolateral (A-to-B), respectively.

### *In Vivo* DMPK Experimental Conditions (PK PBL Cassettes)

#### In-Life Phase

All rodent PK experiments were conducted in accordance with the National Institute of Health regulations of animal care covered in Principles of Laboratory Animal Care and were approved by the Institutional Animal Care and Use Committee (protocols: M2400066-00 and M2500037). Four compounds plus one control were formulated as a solution in ethanol, PEG400, and DMSO (1:3:6 v/v, respectively) at a concentration of 1 mg/mL and administered as a single IV dose (1 mL/kg, 0.2 mg/kg per compound, total 1 mg/kg) to male, Sprague–Dawley rats (*n* = 1) via injection into a surgically implanted jugular vein catheter. Blood samples were collected serially from a surgically implanted carotid artery catheter in each animal over multiple postadministration time points (0.033, 0.117, 0.25, 0.5, 1, 2, 4, 7, and 24 h) into chilled, K_2_EDTA anticoagulant-fortified tubes and immediately placed on wet ice. The blood samples were then centrifuged (1700 rcf, 5 min, 4 °C) to obtain plasma samples, which were stored at −80 °C until analysis by LC-MS/MS.

For determination of the brain over plasma ratio (*K*_p_), the same cassette of compounds plus control was formulated in ethanol, PEG400, and DMSO (1:3:6 v/v, respectively) and administered as a single IV dose (1 mL/kg, 0.2 mg/kg per compound, total 1 mg/kg) to male, Sprague–Dawley rats (*n* = 1) via injection into a surgically implanted jugular vein catheter. At 15 min post dosing, the blood sample was collected terminally into a chilled, K_2_EDTA anticoagulant-fortified tube and immediately placed on wet ice. The blood sample was then centrifuged (1700 rcf, 5 min, 4 °C) to obtain the plasma sample. At the same postadministration time point, the whole-brain sample was obtained by rapid dissection, rinsed with 0.9% saline, and immediately frozen in an individual tissue collection box (dry ice). All brain and plasma samples were stored at −80 °C until analysis by LC-MS/MS.

#### Sample Preparation for Bioanalysis

Plasma samples from the in-life phase of the study were thawed at ambient temperature (benchtop), and then aliquots (20 μL per sample) were transferred to a 96-shallow-well (V-bottom) plate. Matrix-matched quality control (QC) samples and a standard curve of each analyte (1 mg/mL DMSO stock solution) were prepared in blank rat plasma (K_2_EDTA-treated) via serial dilution and transferred (20 μL each) to the plate along with multiple blank plasma samples. MeCN (120 μL) containing IS (10 nM carbamazepine) was added to each well of the plate to precipitate protein. The plate was then centrifuged (4000 rcf, 5 min, ambient temperature), and resulting supernatants (60 μL each) were transferred to a new 96-shallow-well (V-bottom) plate containing an equal volume (60 μL per well) of water (Milli-Q purified). The plate was then sealed in preparation for LC-MS/MS analysis.

Preparation of brain samples was identical to that of plasma samples except for the following modifications. While thawing, brains were weighed (inside their collection boxes using a universal empty collection box tare weight) and then subjected to mechanical homogenization (Mini-BeadBeater, BioSpec Products, Inc., Bartlesville, Oklahoma) in the presence of zirconia/silica beads (1.0 mm) and extraction buffer (isopropanol:water, 7:3, v/v; 3 mL per sample, corrected for postquantitation). Homogenized brain samples were then centrifuged (4000 rcf, 5 min, ambient temperature), and 5 μL of the supernatant was diluted in 15 μL of blank plasma for quantification of the analyte. The plasma standard curve and QCs were used for compound quantitation in brain.

#### LC-MS/MS Analysis

Prepared samples were injected (10 μL each) onto an AB Sciex Triple Quad 4500 mass spectrometer system with an Agilent 1260 Infinity II pump and autosampler. Mass spectrometer conditions are described in Table 1. Quantitation of the analytes was performed via AB Sciex Multiquant software using the raw analyte:IS peak area ratios. The typical detection range was 0.5 ng/mL to ≥5000 ng/mL utilizing a quadratic equation regression with 1/*x* ^2^ weighting.

Correction for dilution of all brain samples (in extraction buffer and subsequently in blank plasma) was performed postquantitation. The corrections for dilution in extraction buffer employed correction factors specific to each brain weight.

### Computational Modeling

#### Homology Modeling of CB_2_

To generate a diverse set of CB_2_ receptor conformations suitable for docking, template-based models were constructed based on all publicly available Class A GPCR structures bound to agonists or allosteric modulators using RosettaCM^54^ for each GPCR template, resulting in an ensemble of receptor conformations representing diverse backbone arrangements relevant to ligand binding. This collection of structures includes structures of CB_2_. Extracellular and intracellular loops were truncated prior to docking, as sampling these flexible regions during docking is not feasible and rigid loop conformations of high-entropy motifs could introduce artifacts. This ensemble of CB_2_ models served as the structural platform for subsequent docking.

#### Molecular Docking

Ligand conformers were generated with BCL::Conf (BCL v4.3.1),^55^ producing up to 250 unique conformers using a SymmetryRMSD tolerance of 0.25 Å. The best conformers were initialized to a CB_2_ putative pocket by performing property-based flexible alignment to an experimentally resolved small-molecule ligand or lipid component using BCL::MolAlign. In total, docking simulations were initiated from each putative binding region, resulting in approximately 470 independent docking runs with 2000 candidate poses generated per run for a total of approximately 940,000 candidate docking poses. Backbone conformations were allowed to exchange between templates during docking, optimizing predicted binding modes over the ensemble. Candidate poses were ranked using the Franklin2019 membrane score function in Rosetta. Backbone conformations based on different templates were allowed to exchange during docking to optimize predicted ligand binding modes across the ensemble. All receptor conformational exchanges occurred during sampling with the Transform mover in Rosetta, while all side-chain packing and refinement occurred on the best scoring single receptor conformation for each docking run.

#### Molecular Dynamics Simulations

All molecular dynamics (MD) simulations were performed using the AMBER 24 software package.^56^ The CB_2_ receptor was simulated in two distinct conformers bound to VU6077967 to assess the relative stability of different docking poses.

Protein–ligand complexes were parametrized using the ff19SB protein force field,^57^ Lipid21 lipid force field,^58^ GAFF2 ligand force field, and the OPC water model.^59^ Each system was embedded in a heterogeneous lipid bilayer composed of 40% POPC, 25% POPE, 10% POPS, 5% PSM, and 20% cholesterol and solvated with OPC water molecules using the packmol-memgen program in AmberTools.^60^ Systems were neutralized and adjusted to a final salt concentration of 150 mM NaCl.^61^ Ligand parameters for VU6077967 were generated using a multistage protocol that combined molecular mechanics (MM) minimization (MMFF94s) followed by quantum mechanical (QM) geometry optimization (ωB97XD/6-311G­(d,p)) and AM1-BCC atomic charge fitting.

Minimizations were performed in three stages (solvent with the protein restrained, protein with the solvent restrained, and the full system unrestrained) using a 12.0 Å nonbonded cutoff and periodic boundary conditions (ntb = 1). Each stage used 1000 total minimization steps (500 steepest-descent followed by 500 conjugate-gradient). Bonds involving hydrogen were constrained (SHAKE, ntc = 1).^62^

Equilibration involved heating to 100 K under the canonical (NVT) ensemble for 1.0 ns, followed by heating to 310 K under the isothermal–isobaric (NPT) ensemble at 1 bar using a Monte Carlo barostat and semi-isotropic scaling over an additional 2.0 ns. Hydrogen mass repartitioning was applied to nonsolvent atoms, enabling a 4.0 fs production time step. A Langevin thermostat with a collision frequency of 1.0 ps^–1^ was used during equilibration and production. The total production simulation time was 500 ns, and nine independent replicates were performed for each docking pose. This resulted in a total simulation time of 9000 ns.

## Supplementary Material







## Data Availability

We will freely provide our CB_2_ template-based modeling conformational ensemble that we used for docking, as well as our top 5 best docked poses and all 18 of our MD simulation trajectories. Computational modeling data will be deposited in a publicly accessible Zenodo repository.

## Notes

Additional compound characterization for **11d** (VU6077967) and molecular pharmacology data, and supplemental data for PK PBL cassettes and computational modeling (PDF)
Molecular formula strings of tested compounds (CSV)
CB_2_ binding pose 2 for VU6077967 (**11d**) (PDB)

## Abbreviations

2-AG: 2-arachidonoyl-glycerol
Boc: *tert*-butyloxycarbonyl
cAMP: cyclic adenosine monophosphate
CB_1_: cannabinoid receptor 1
CB_2_: cannabinoid receptor 2
Cbz: benzyloxycarbonyl
CL_p_: plasma clearance
CNS: central nervous system
EC: endocannabinoid
FFAR3: free fatty acid receptor 3
GIRK: G protein-coupled inwardly rectifying potassium channel
GPCR: G protein-coupled receptor
HEK: human embryonic kidney
HIV: human immunodeficiency virus
HTS: high-throughput screen
M_1_: muscarinic acetylcholine receptor 1
M_4_: muscarinic acetylcholine receptor 4
MD: molecular dynamics
MDCKII-MDR1: Madin-Darby canine kidney cell line transfected by human MDR1 gene
PAM: positive allosteric modulator
PBL: plasma-brain levels
P-gp: P-glycoprotein
PI: phosphatidylinositol
PK: pharmacokinetic
PPB: plasma protein binding
RMSD: root-mean-square deviation
SAR: structure–activity relationship
SD: Sprague–Dawley rat
*t*_1/2_: half-life
THC: (−)-trans-Δ^9^-tetrahydrocannabinol
*V*_ss_: volume of distribution at steady state
