# Supplementary material for: Drug Repurposing: Conversion of the Peripherally Restricted HIV Protease Inhibitor Amprenavir to Potent, Selective, and CNS-Penetrant Agonists for the Cannabinoid Receptor 2
Source: J Med Chem. 2026 Feb 5;69(4):4187–207. doi: 10.1021/acs.jmedchem.5c02796 (PMC12951455; doi:10.1021/acs.jmedchem.5c02796)
Supplement: Supplementary file 1 [file jm5c02796_si_001.pdf]

Supporting Information

Drug Repurposing: Conversion of the Peripherally Restricted HIV Protease Inhibitor Amprenavir to Potent, Selective, and CNS-Penetrant Agonists for the Cannabinoid Receptor 2

Daniel H. Haymer,[a,b,^] Renn A. Duncan, [a,b,^] Alice L. Rodriguez,[a,b] Allie Han,[a,b] Richard J. Lindsay,[b,c], N. Kithmini Wijesiri,[b,c] Analisa Thompson Gray,[a,b] Srinivasan Krishnan,[a,b] Aidong Qi, [a,b] Benjamin P. Brown,[b,c] Olivier Boutaud,[a,b] Darren W. Engers,[a,b] Carrie K. Jones,[a,b,d,h] Colleen M. Niswender,[a,b,d,e,f,h*] Craig W. Lindsley,[a,b,g,h*] Aaron M. Bender [a,b*]

a. Warren Center for Neuroscience Drug Discovery, Vanderbilt University, Nashville, Tennessee 37232, United States
b. Department of Pharmacology, Vanderbilt University, Nashville, Tennessee 37232, United States
c. Center for AI in Protein Dynamics, Vanderbilt University, Nashville, Tennessee 37232, United States
d. Vanderbilt Brain Institute, Vanderbilt University, Nashville, Tennessee 37232 United States
e. Vanderbilt Kennedy Center, Vanderbilt University Medical Center, Nashville, Tennessee 37232, United States
f. Vanderbilt Institute of Chemical Biology, Vanderbilt University, Nashville, Tennessee 37232, United States
g. Department of Chemistry, Vanderbilt University, Nashville, Tennessee 37232, United States
h. Vanderbilt Institute for Therapeutic Advances, Vanderbilt University, Nashville, Tennessee 37232, United States

Corresponding author emails: *craig.lindsley@vanderbilt.edu, colleen.niswender@vanderbilt.edu, aaron.bender@vanderbilt.edu

Supplemental Figure 1. LCMS Trace for Key Analog 11d **S2**

Supplemental Figure 2. rCB$_1$ Selectivity for Selected Analogs **S3**

Supplemental Figure 3. Potency Correlation in the Presence and Absence of 2-AG **S4**

Supplemental Table 1. LC-MS/MS Conditions for PK PBL Cassettes **S5**

Supplemental Figure 4. Representative Standard Curve for CB$_2$ PAM Analysis **S6**

Supplemental Computational Figures 5 and 6 **S6**

Supplemental Figure 1. LCMS Trace for Key Analog 11d

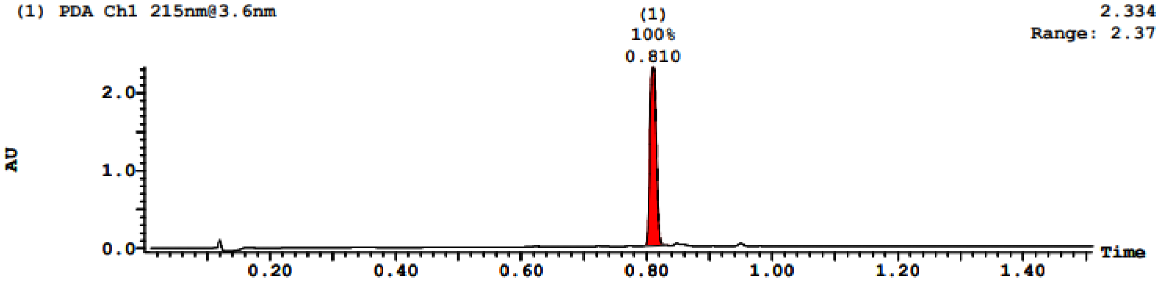

Peak Number	Time	Area %Total	BPM	Mass Found
1	0.810	100.00	505	Not Found

Peak ID	Time
1	0.810

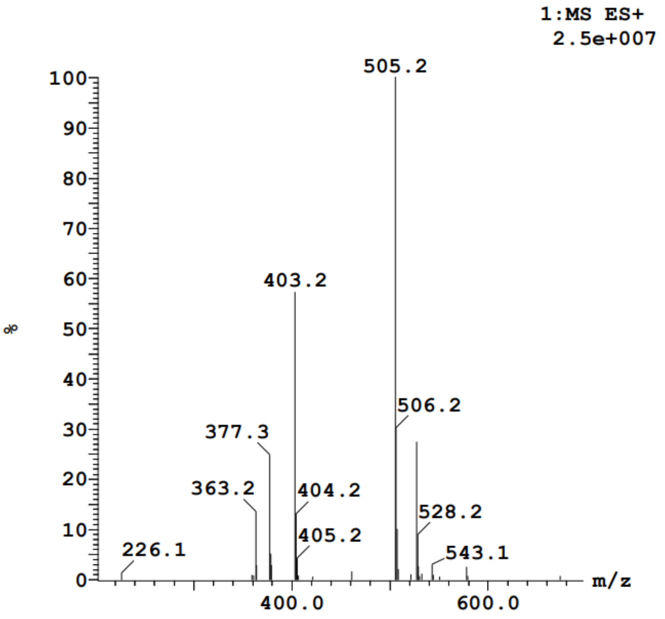

Supplemental Figure 2. rCB_1 Selectivity for Selected Analogs[a]

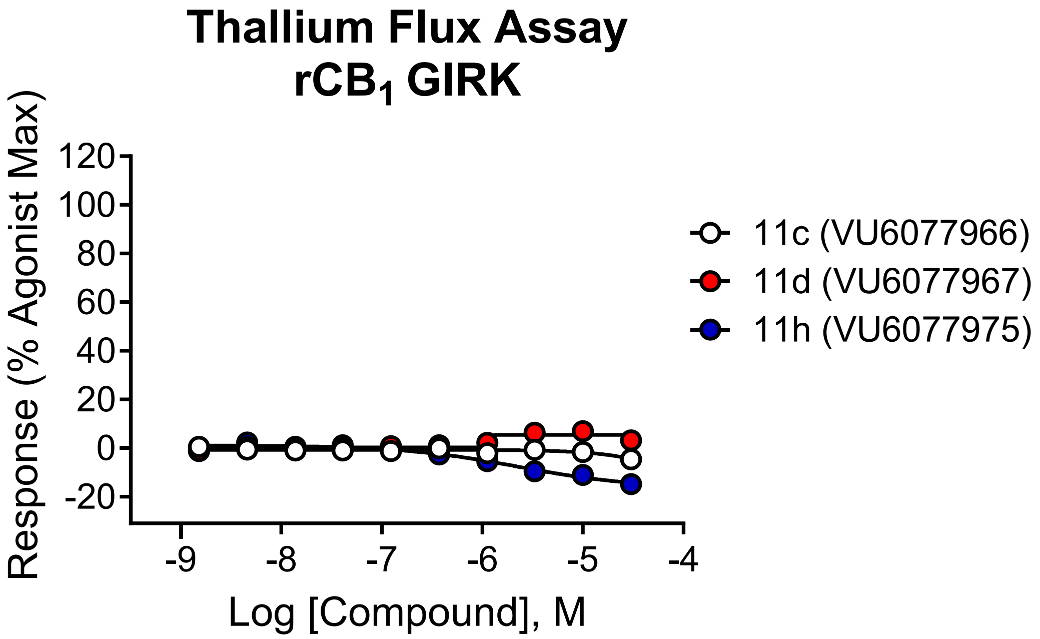

a. Amprenavir analogs are selective for rCB_2 versus rCB_1. Concentration-response curves in thallium flux assays for **11c**, **11d**, and **11h** in the absence of agonist (2-AG) are shown. Data represent one experiment run in triplicate. Data are plotted as a percentage of maximal 2-AG response.

Supplemental Figure 3. Potency Correlation in the Presence and Absence of 2-AG[a]

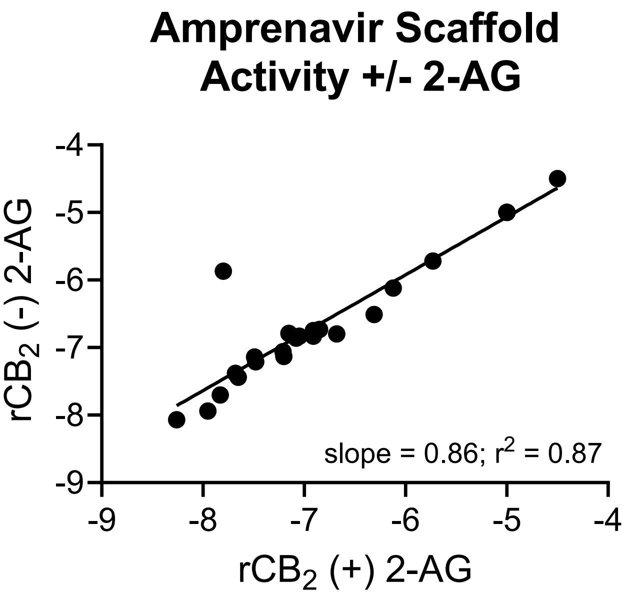

	(+) 2-AG					(-) 2-AG				
Cmpd#	rCB2 pEC50 AVG	rCB2 pEC50 SEM	rCB2 EC50 AVG (nM)	rCB2 % 2-AG Max AVE	rCB2 % 2-AG Max SEM	rCB2 pEC50 AVG	rCB2 pEC50 SEM	rCB2 EC50 AVG (nM)	rCB2 % 2-AG Max AVE	rCB2 % 2-AG Max SEM
5	6.12	0.06	760	49	2	6.12	0.06	969	55	2
11a	6.31	0.10	485	38	5	6.51*	0.06	306	48	1
11b	7.20	0.05	63.0	58	4	7.13	0.09	74.4	94	6
11c	7.65	0.15	22.5	53	4	7.44	0.16	36.0	76	4
11d	8.26	0.04	5.45	77	3	8.07	0.21	8.6	119	14
11e	< 5.0		>10000	-10	8	< 5.0		>10000	-30	13
11f	6.91	0.10	123	47	4	6.75	0.28	176	58	3
11g	< 5.0		>10000	-44	4	< 5.0		>10000	-95	13
11h	7.15	0.01	70.5	65	2	6.79	0.16	161	88	6
11i	7.48	0.12	32.9	75	3	7.21	0.03	61.4	105	2
11j	7.21	0.08	61.5	63	3	7.06	0.05	87.8	84	3
14a	7.95	0.09	11.2	77	1	7.94	0.16	11.6	104	7
14b	7.05	0.14	88.4	69	2	6.83	0.05	148	82	2
14c	7.68	0.08	21.1	74	2	7.38	0.10	41.4	117	8
14d	5.73	0.16	1849	58	3	5.72	0.09	1917	79	7
14e	7.49	0.14	32.6	71	5	7.14	0.18	71.9	100	6
14f	7.83	0.15	14.9	79	8	7.70	0.07	19.9	120	14
14g	7.80	0.07	15.7	77	2	5.87	0.09	1334	131	8
14h	7.08	0.12	82.8	72	1	6.86	0.08	138	93	4
14i	< 5.0		>10000	-10	2	< 5.0		>10000	-49	16
21a	< 5.0		>10000	-32	3	< 5.0*		>10000	-85	4
21b	6.91	0.15	122	35	3	6.83*	0.18	146.3	36	9
21c	Inactive					Inactive				
21d	6.85	0.10	140	42	5	6.73	0.16	185	43	1
21e	6.68	0.12	211	43	3	6.80	0.15	160	42	3

a. There is a strong correlation in potency values when comparing rCB_2 activity in the presence and absence of submaximal 2-AG. The $\log(EC_{50})$ for a subset of compounds in the amprenavir scaffold were plotted in the presence (x-axis) or absence (y-axis) of 2-AG and a linear regression was performed. $r^2 = 0.87$, $p < 0.0001$. EC_{50} values are calculated from the mean pEC_{50} values of at least three independent experiments run in duplicate or triplicate unless otherwise noted (*indicates two independent experiments).

Supplemental Table 1. LC-MS/MS Conditions for PK PBL Cassettes

Injection volume	10 µL	
Mobile phase A	0.5% Formic Acid in Water	
Mobile phase B	0.5% Formic Acid in Acetonitrile	
Flowrate	0.5 mL/min	
Gradient	Time	% Mobile Phase B
	0.0	5
	0.2	5
	0.8	95
	1.5	95
	1.7	5
	2.7	Stop
Column	Fortis C18 (50 x 3.0 mm, 3 µm)	
Data collection and analysis software/version	Analyst v. 1.7.1	
Ionization mode	Positive Electrospray	
Collision gas (psi)	9	
Curtain gas (psi)	40	
GS1 (psi)	40	
GS2 (psi)	40	
Capillary voltage (V)	5500	
Source TurboIonSpray® temp. (°C)	500	
MRM mass transitions (Da):		
I.S. (Carbamazepine)	237.0/193.9 (CE: 25, DP: 96, EP: 10, CXP: 8)	
11c	505.2/403.2 (CE: 18, DP: 20, EP: 10, CXP: 13)	
11h	471.2/258.1 (CE: 28, DP: 40, EP: 10, CXP: 10)	
11i	459.2/246.1 (CE: 26, DP: 40, EP: 10, CXP: 10)	
11j	473.2/260.1 (CE: 28, DP: 40, EP: 10, CXP: 10)	
14b	540.1/438.2 (CE: 19, DP: 20, EP: 10, CXP: 13)	
21d	457.2/244.1 (CE: 45, DP: 40, EP: 10, CXP: 35)	
21e	445.3/232.2 (CE: 41, DP: 40, EP: 10, CXP: 35)	

Supplemental Figure 4. Representative Standard Curve for CB_2 PAM Analysis by LC-MS/MS

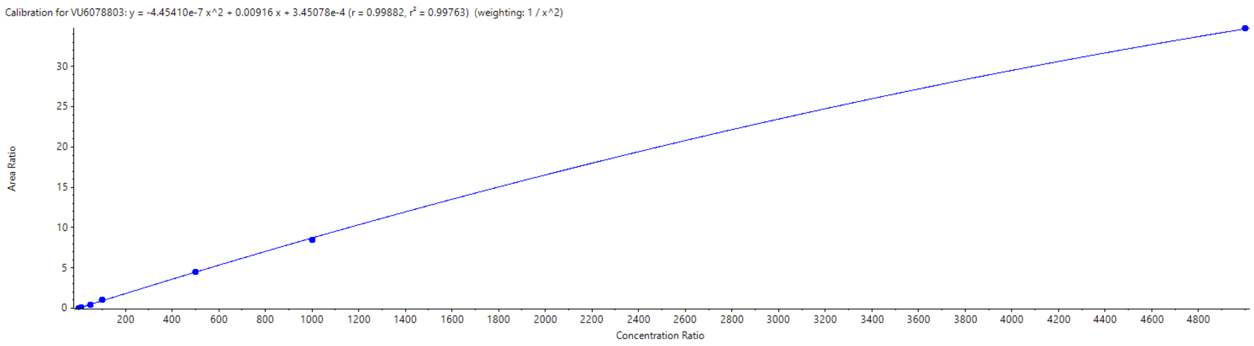

Supplemental Computational Figures

Supplemental Figure 5. Predicted Docking Poses of VU6077967 (11d) to CB_2[a]

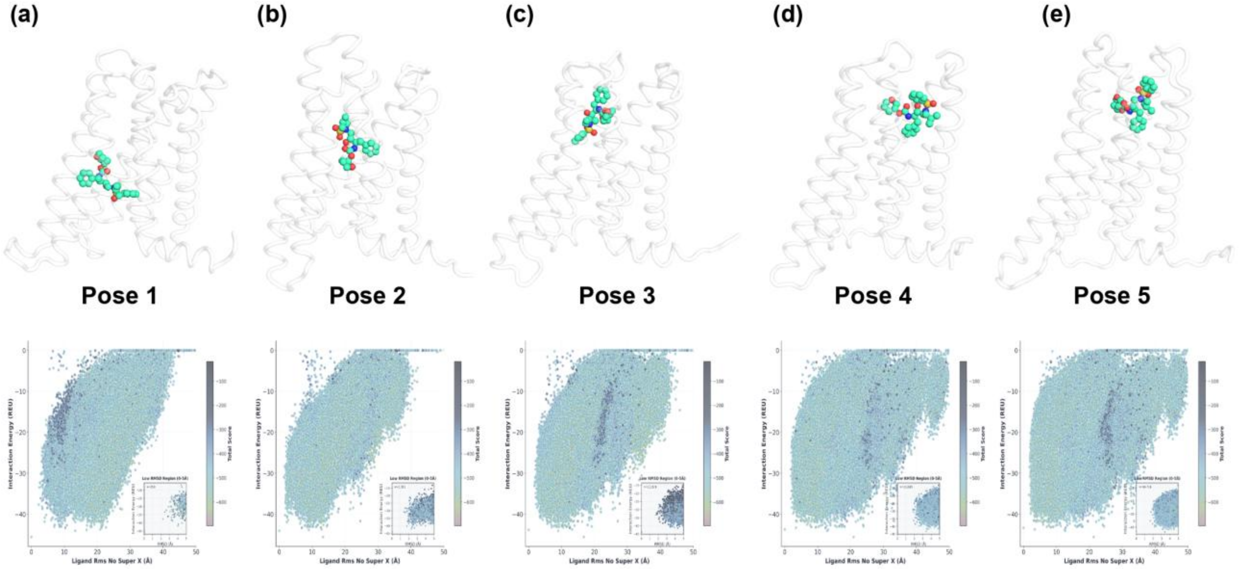

a. Docking score vs. RMSD plot for VU6077967 where RMSDs are computed against the respective shown docking poses.

Supplemental Figure 6. Comparison of FFAR3 Agonist AR420626 Binding Mode to FFAR3 with Predicted Pose 1 of VU6077967 (11d) to CB_2[a]

(a) **(b)**

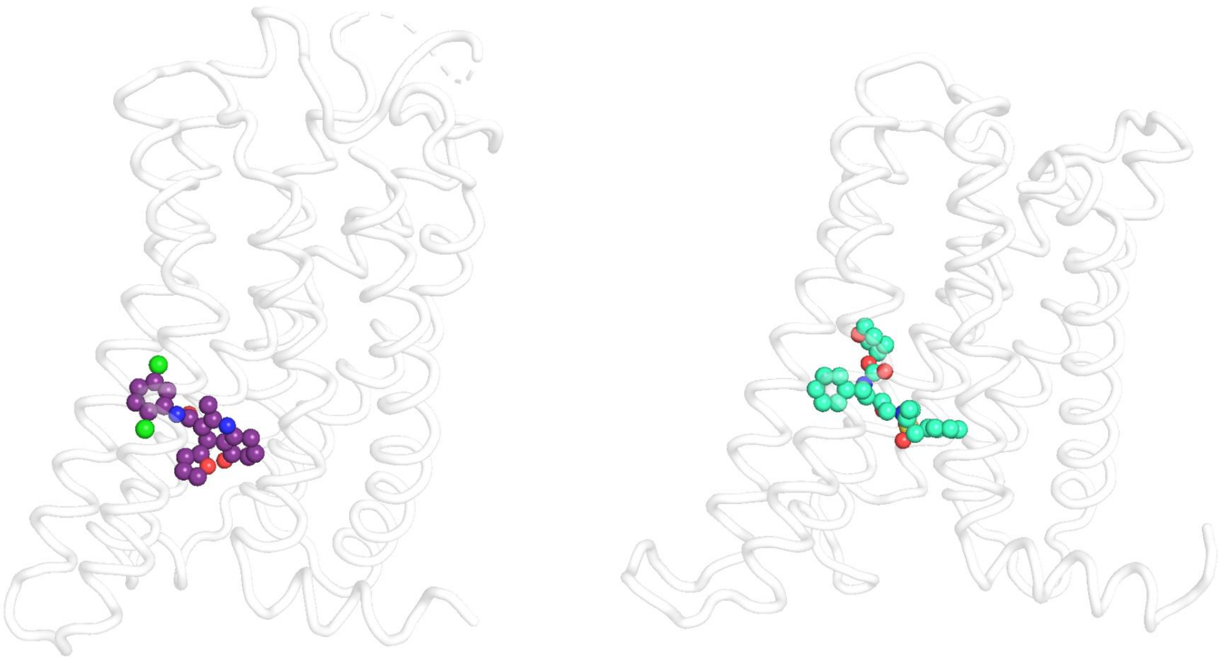

AR420626 **VU6077967 p1**

a. (a) Experimentally determined binding mode of AR420626 to FFAR3 (PDB ID 8J20). (b) Predicted pose 1 of VU6077967 to CB_2.
